# Eradicating the germs of pauperism: the political economy of the British sanitary movement in the 1830s and 1840s

**DOI:** 10.1590/S0104-59702024000100063en

**Published:** 2024-12-16

**Authors:** Daniel Schneider Bastos

**Affiliations:** i Post-doctoral fellow, Graduate Program in History/Universidade Federal Fluminense. Niterói – RJ – Brazil danielbastos@id.uff.br

**Keywords:** Nineteenth-century history, Public health, Industrial development, Capitalism, Sanitary Movement

## Abstract

The article addresses the British sanitary movement in the 1830s and 1840s, analyzing the ideology that permeated official efforts to promote public health. The sources used consist primarily of the inquiries carried out by royal commissions into the state of sanitation in towns and cities. The main argument is that the ideological assumptions underpinning these inquiries can be understood as part of a political economy of public health, within which tensions can be observed arising from the contradictions between a liberal perspective and the need for greater intervention on the part of the government.

In social history, the period of the First Industrial Revolution, which took place in Great Britain between approximately the 1780s and 1840s, is also known as the Age of Reform or the Age of Improvement. Britain’s failure in handling the crisis in the Thirteen Colonies triggered a wave of dissatisfaction towards the government, which was deemed corrupt, costly, and inefficient, leading different social groups to unite under the banner of a parliamentary reform capable of making the government more transparent and responsive to the demands of society. However, the Age of Reform is a term whose meaning goes beyond just political and parliamentary issues, insofar as the reform movement came to have other meanings attached to it. These included the idea of institutional reform, which called for improved services and institutions, as well as moral and religious reform, marked by a desire to make amends for the vices of public and private life, a consequence of the revival of evangelicalism^1^ in the eighteenth century (Innes, 2004).

The 1830s and 1840s form a particularly important juncture in the implementation of reforms. There was an electoral reform in 1832, followed in 1835 by a municipal corporations reform,^2^ which made the workings of politics more dynamic and introduced new liberal conceptions to the State. There were decades of intense public debate as the impacts of industrialization and urbanization on people’s lives increasingly shaped public opinion. One of the issues on the agenda was sanitation, for which purpose repeated official investigations were conducted, whose political undertones were far more complex than the putative objectivity of sanitation measures might initially suggest.

This article focuses on the ideological dimension of the British sanitary reform movement in the 1830s and 1840s, specifically the changes associated with Edwin Chadwick, a well-known reformer who was active on a range of institutional fronts. The primary sources upon which the analysis is based consist largely of the reports related to the topic published in the 1830s and 1840s by different royal commissions. Of particular note are the publications of three commissions: the Poor Law Commission (active between 1834 and 1847), particularly the supplementary reports relating to the fever problem, included in the fourth report, from 1838, and the health report by Chadwick, published in 1842, which was supplemented the following year; the Health of Towns Commission (1843 to 1848), whose two reports were published in 1844 and 1845; and the Health of the Metropolis Commission (1847 to 1850), which published two reports in 1848. Reports by other royal commissions not inquiring directly into public health, but with findings that were in some way related to it, were also included in the analysis. Such was the case of the reports of the commissions set up to investigate children’s employment in factories (1833), the operation of the Poor Laws (1833 to 1834), and the working conditions of hand-loom weavers (1837 to 1841). All these documents, which are part of the Parliamentary Papers, are available for reference purposes in hard and digital format.^3^

The discussion of the commissions’ work is based on the content of the main reports^4^ delivered to Parliament. The appendices, which are generally longer than the final reports, including initial descriptions of local or regional scenarios, responses to questionnaires, letters of instruction to commission members, and other materials, were not consulted for this research. Similarly, none of the archives of internal correspondence or bureaucratic papers issued by the commissioners in the course of their duties were analyzed.

The inclusion of these sources would fill gaps on certain aspects of the royal commissions and their actors that are not explicit in the final reports. However, including them would make it unfeasible to draw on the work of multiple commissions and thus to make the intended cross-sectional analysis. Even if we admit this decision as a limitation to the chosen methodology, it would be fair to counterargue that this article does not aim to draw an accurate picture of the state of public health in Great Britain or the sanitary movement in general. As pointed out by McLean (2006, p.x-xi), any such reconstruction of a trajectory of this nature by focusing on parliamentary acts and central institutions, such as royal commissions, would risk overlooking the influence of local initiatives, where the political game had more direct consequences for British people’s daily lives, not least because the resources to finance sanitation infrastructure came from local coffers.

What this article proposes to do is to trace the paradigms that permeate the work of the aforementioned royal commissions with a view to observing how much the worldview underpinning one current in the sanitary movement, which enjoyed political momentum at the time, had a liberal bias. The discourse used in their final reports, containing a synthesis of the work done by the members of each commission and prepared with an eye to advancing certain political agendas, is a prime example for the analysis of this articulation from the perspective of the sanitarians themselves. Based on these reports, the analysis discusses how liberalism and other associated currents affected the understanding of sanitation and public health that guided the reformers’ actions, even while admitting a degree of contradiction between these ideological constraints and certain practical considerations inherent to the materiality of the demands imposed by industrial capitalism and the new urban geography. It is argued that by observing such contradictions, it is possible to trace how the political economy was manifested in the sanitary reform campaign, in close liaison with other reformist initiatives during the period studied.

## A trajectory of the British sanitary movement until the Public Health Act of 1848

The sanitary movement took place within a complex web of social tensions, which had to do with a general decline in health indicators in towns and cities, social movements for better working conditions, the emergence of Chartism as a mass movement, efforts to standardize and centralize the management of pauperism through the Poor Laws, a trend towards the employment of qualified staff in public administrations, among other factors that contributed to the political tensions of the 1830s and 1840s. While recognizing this intricate scenario and the multiple interconnections between these different phenomena, the influence of more episodic facts on the development of the public health agenda should not be overlooked.

One decisive event was the cholera epidemic in England, which began in October 1831, when it was introduced through the port of Sunderland. A few months earlier, in June 1831, the Central Board of Health had been convened. Made up primarily of members of the Royal College of Physicians, in London, this board was called on at times of health emergencies to advise the Privy Council.^5^ The first Central Board of Health had been formed in 1805, when the Privy Council needed a group of experts to help develop measures to prevent yellow fever from reaching the United Kingdom. It had been dissolved a year later and had not been convened since. In the months following its revival in 1831, the board was active in fulfilling its function, but it wielded little effective power beyond advising the government. Powers to take measures to fight cholera were in the hands of local boards, usually covering a single parish or town. Indeed, there had been local health boards in some cities since the late eighteenth century. The number of such boards multiplied in response to the epidemic in 1831 and 1832, when around 1,200 new ones were created. They subsequently gained more robust legislative recognition in 1848, with the passing of the Public Health Act (Brockington, 1961).

In December 1832, after the end of the epidemic, the Central Board of Health was disbanded. The legislation that had strengthened the powers of local bodies to combat the disease expired, leaving a hiatus. The prevailing mindset was that public health interventions were to be taken only in the event of an emergency, not as part of a permanent, prevention-oriented policy.

While the institutional response to cholera may have been weak, the disease still left a strong impression on the sanitary awareness of the British population. The epidemic highlighted not only the deterioration of health conditions in towns and cities, but also the social nature of this deterioration. Observers equipped with statistical data noted how much more lethal cholera was in working-class neighborhoods than it was in wealthier districts. As reported by the Spanish physician Mateo Seoane (1832, p.10), a pioneer of sanitary measures in Spain who was sent to England to gather information on cholera after the start of the epidemic,

it is also of note that almost all the people who were taken ill in Sunderland were from the part of the city where poor people live, since here, as in all other parts of England, cholera has prevailed mainly in the poorest abodes, and only when it has spread widely has it been seen to affect the wealthy. It is curious to observe the predilection of this terrible malaise for filthy, cramped, and poorly ventilated spaces, and the poor who dwell in them: this fact had been observed in Warsaw, Berlin, Hamburg and other places, and has been confirmed most patently in England.^6^

Given the context of the early 1830s, marked by social movements calling for political reform, the combative action of trade unions (only recently made legal), and much more public spending aimed at addressing pauperism than at the beginning of the century, it was as if the appearance of cholera confirmed the worst fears of those who had been decrying the dangerous decline in the physical and moral condition of the working classes. With a mixture of curiosity, disbelief, and dismay, the government, ruling classes, and middle classes seemed to suddenly become aware that they coexisted, in the very same country, with a mass of working men and women whose habits and living conditions seemed as foreign to them as those of the native peoples from the remotest corners of the Empire. In its 1848 report, the royal commission responsible for inquiring into the state of public health in London referred to the arrival of cholera in the following terms:

At that time not only had no knowledge been acquired by experience of the true character of this disease, but nothing was known of the real condition of the classes which proved to be its first and easy victims, nor of the state of the localities in which they lived. The metropolis of the poor has nothing in common with the metropolis of the rich, and as the map of London exhibits no trace of the lanes and alleys of the poor, so the very names of these places would at that time have sounded as strange to the inhabitants of our great thoroughfares and squares as the names of the streets of a foreign country (Great Britain, 1848a, p.6).

Although medical and health authorities were aware that the tens of thousands of deaths caused by the epidemic was on a par with the number of lives claimed by diseases such as smallpox, typhus, influenza, diphtheria, tuberculosis, and more common diseases of the digestive system, cholera proved to be more socially disruptive. It had not been naturalized like other illnesses and caused social hysteria whenever it sprung up. Causing violent diarrhea (something that quite offended the prevailing sense of modesty) and capable of leading to death within 12 hours of the first symptoms, it haunted the collective imagination. The inability of medicine to find an effective treatment only exacerbated the panic associated with cholera (Evans, 1999, p.159-173).

The cholera epidemic coincided with an intensification of social movements calling for parliamentary reform, which finally came about with the Reform Act of 1832. The reformist impetus paved the way for the Whig party^7^ to return to power after decades of Tory rule. One of the new government’s first priorities was to overhaul the Poor Laws^8^ in order to reduce the tax burden of pauperism and tackle what was perceived as a state of generalized immorality induced by the ease with which the poor were supposedly able to obtain parish relief. Since the late 1700s, rapid demographic expansion, inelastic labor supply in rural parts, and inflation caused by the wars with France had resulted in repeated tax hikes with a view to managing pauperism. Hostility in political and intellectual circles against the Poor Laws grew, as exemplified by the work of Malthus (1998). The Swing Riots of 1830, a wave of civil disturbances in the countryside in which impoverished rural laborers destroyed machinery and properties, secured the support of the landed gentry for a reform project that lent the parish system a disciplinary edge.

In 1833, a royal commission was appointed to investigate how the Poor Law was working in England and Wales and how to improve it. It published its final report in 1834 (Great Britain, 1834), and in that same year Parliament passed an amendment act, based on the commission’s recommendations, making what became known as the new Poor Law. Under the new legislation, English and Welsh parishes were grouped into parish unions, each responsible for financing the construction and maintenance of at least one workhouse. If the poor wished to receive relief, they were required to voluntarily enter the workhouse – an institution designed to be as unpleasant as possible – where they had to earn their keep. The thinking behind this was that anyone who sought relief under the Poor Laws should be worse off than those employed in the worst jobs available on the market, ensuring that only those in truly desperate straits would turn to the state for assistance (Great Britain, 1834, p.127-150).

Despite being based on distorted assumptions about the social reality, the reform was effective in reducing expenditure on pauperism.^9^ Furthermore – and contrary to the discourse that inspired its creation – the new Poor Law actually, over time, proved to be an important milestone on the road towards the creation of public health services for the working population, since the workhouses had doctors on their staff and infirmaries as part of their facilities. Over the following three decades, these institutions became increasingly associated with the provision of health services, with the distinction between assisted poor and patient becoming increasingly hazy.^10^

Tasked with directing the practical implementation of the new legislation, the 1834 amendment act set up the Poor Law Commission, a royal commission with powers to act as the national authority for matters relating to the reformed system, to which the parish unions had to report. Its function was to ensure that the new guidelines for pauperism were adopted by local authorities in as standardized a manner as possible. The history of this commission is directly linked to the direction of British public health. It was through its actions that the state of health in the country started to be studied more systematically by the government. In its fourth annual report, from 1838, the commissioners included two supplementary reports on the causes of fever among workers in the Metropolis (London), penned by doctors from the commission. Both writings pointed to the need to combat miasmas, understood to be harmful gases that emanated from putrefied matter and caused disease.

The first of these supplementary reports, written by Neil Arnott and James Kay, lists six measures to extinguish “miasmatic exhalations” in the city, which were reiterated by subsequent commissions: to build adequately lined sewage channels connecting houses and streets; to ensure an abundant supply of water for household use and the cleaning of public rights of way; to hire street sweepers to remove any dirt that could not be removed by water; to improve the circulation of air by widening roads, creating squares, and strategically demolishing certain buildings; to move activities and environments believed to cause miasmas, such as cemeteries, slaughterhouses, livestock farms, and gas factories, away from the busiest areas; to avoid overcrowding in cheap inns (Great Britain, 1838, p.103-129). Thomas Southwood Smith, who wrote the other supplementary report, agreed on the importance of preventing the production and concentration of miasmas. He also advocated setting up cholera hospitals so that people with the disease would not end up in the workhouse, where they might transmit the disease to others (Great Britain, 1838, p.129-151).

The reports prompted the secretary of the Poor Law Commission, Edwin Chadwick, to delve deeper into the sanitation agenda. Chadwick had been one of the commissioners responsible for writing the report that led to the 1834 reform. Frustrated at not having been named one the three main commissioners appointed with the new legislation, he took advantage of his social contacts with some leading political figures to persuade Parliament to call for a national investigation into the health of the nation. His wish was granted in 1839, when he was given leave to step down as secretary to dedicate himself to this new project.

The result of the new inquiry was published in 1842 and went on to become a bestseller, selling more than one hundred thousand copies. Its supplementary report, concerning burial practices in cities, came out the following year. Its publication coincided with the height of what historians have called the “age of commissions” or the “age of Blue Books,”^11^ when speaking of the second quarter of the nineteenth century, in reference to the great number of inquiries into social conditions and the way their reports captured the public imagination (Hilton, 2006, p.602-604; Briggs, 2014, p.238). Some inquiries were undertaken by individuals interested in communicating their point of view on issues they felt deserved greater attention. Meanwhile, the government, which had the power to create parliamentary committees to carry out investigations, increasingly relied upon the work of royal commissions of inquiry. Appointed by the Home Office, these commissions could be made up of anybody the ministers deemed fit to contribute to the matter at hand.

Historically, these commissions sparked some controversy, given that they operated in parallel to Parliament, stemming from the royal prerogative to appoint agents for data collection tasks. The increasing use of such commissions in the nineteenth century was driven by a general understanding that their members were at the service of the government, not the Crown, and as such they did not constitute an intrusion by monarchical authority. But their increased use was also a corollary of the desire to have more qualified individuals responsible for inquiring into important topics and also ones who may not be swayed so greatly by political disputes, at a time when the bipartisan dynamic of Parliament and local elections was becoming crystallized (Lockwood, 1967).

A prosopography of the men who served on the royal commissions set up to investigate topics of social interest in the 1830s and 1840s reveals them to come from quite similar stock. There was a disproportionate representation of men from traditional landed classes, known as the landed gentry, but there were also men from families engaged in international trade, professional men from different fields, and a few industrialists and lesser tradesmen. The traditional careers of law and medicine^12^ were the most common among the commissioners, but there were a great many other occupations that point to the growing specialization and professionalization of different fields, including engineers, geologists, chemists, paleontologists, and economists, in addition to clergymen, military men, and businessmen. Some commissioners were self-made men who had risen in society through business acumen or professional prestige. When commissions were set up, an effort was made to ensure a balance of the criteria of merit and competence with those of lineage and tradition. In this way, the commissions counted on the skills and competencies needed to carry out more skilled, scientific inquiries, but without giving up the political capital needed to legitimize their work and facilitate the collaboration of local authorities (Bastos, 2023, p.99-161). Chadwick himself came from a lower-middle class family: his father was a radical journalist. His rise in public life was due in large part to his proximity to the philosopher and jurist Jeremy Bentham, whose utilitarian philosophy was a determining influence among the social reformers of his day.

When Chadwick’s report came out in 1842, it caused such an outcry that a royal commission of inquiry was appointed, in 1843, to scrutinize the state of health in the towns and cities of England and Wales – a task that took until 1848 and gave rise to two main reports (Great Britain, 1844, 1845). Another commission was appointed in 1847 with the specific task of investigating conditions in London; its work lasted until the following year and also gave rise to two reports (Great Britain, 1848a, 1848b). Both initiatives confirmed what Chadwick had reported. Indeed, Chadwick himself was a member of the London commission and also kept a close eye on the work of the commission covering England and Wales. The crowning achievement of Chadwick and this approach to sanitation as an official policy came with the passing of the Public Health Act of 1848, which established the General Board of Health, with Chadwick and two other men at the helm. The following year, he took over as director of the newly created Metropolitan Commission of Sewers, making him the supreme sanitation authority in the capital city.

Chadwick’s heyday was brief. While in theory the General Board of Health had extensive powers to intervene through local councils, it could not enforce their creation in places where there was none. At its head, Chadwick repeatedly complained of a lack of funding, personnel, and collaboration by other public institutions. At the same time, the limits of his sanitary theory became clear when the decision was taken to have London’s sewage channels discharge their contents straight into the Thames, which aggravated the spread of cholera: new epidemics broke out in England in 1848-1849, 1853-1854, and 1866. Friction with local authorities, quarrels with water companies (which had a powerful lobby in Parliament, where many of their shareholders sat), and a failure to prevent the return of the very disease that had propelled him to prominence all contributed to Chadwick’s rapid decline and ultimately forced him to resign in 1854. The General Board of Health was dissolved in 1858, and its functions were transferred to the Privy Council. The relationship between national and local authorities and the degree to which government should intervene in private matters would remain subjects of debate as new strategies for the sanitation problem were designed.

In the historiography of this period, debate abounds over the nature of the reform movement, including sanitation. Much of this debate revolves around disagreements over whether the reforms were ad hoc measures aimed at providing objective, sectorized solutions to specific social ills, or whether there were more ideological motivations at play, in which case interest has focused on identifying evidence of elements in the reformers’ actions that fit within a broader agenda, imbued with the political and economic expectations of groups that were gaining power as the Industrial Revolution progressed. Authors critical of the latter approach tend to denounce its supposedly “conspiratorial” nature and failure to take account of the nuances within the pro-reform groupings, reducing them to a social readjustment plan orchestrated by the bourgeoisie and its associates (Fraser, 1984, p.xxii-xxx).

This article avoids this dichotomy by proposing a political economy analysis of the sanitary movement. The aim is to recognize the pragmaticism of the reformers’ efforts, without giving up the idea that their actions converged in directions that reveal the existence of ideological assumptions embedded in this movement. The effective exercise of reform, affecting as it does material issues of everyday life, inevitably involves a degree of pragmatism. Nonetheless, the dissonances that occur between the theoretical and practical planes expose contradictions that bring the contours of the ideology into relief as it is tested by the challenges imposed by social reality and its management. Two dilemmas that stand out in sanitation will guide the remainder of this study: first, the conflict between centralizing and interventionist drives in the context of the defense of a *laissez-faire* approach; and second, the challenge of reconciling the socio-environmental aspect of public health with a moral and individualizing reading of this same topic.

## Intervention and centralization in a *laissez-faire* society

Among the liberals and conservatives aligned with the two hegemonic parties, there was a consensus around the defense of private property and capitalist relations. In an English tradition where parishes and corporations had autonomy to run local affairs, under the management of officials and councils controlled by property owners or their favorites, this position could represent a challenge to any interventionist measure. Neither political economy nor utilitarianism – both philosophies that influenced liberal reformism – ever presented liberalism as a synonym for absolute non-intervention. Still, the line separating legitimate regulation from intolerable violation was a constant cause of tension between property owners and reformers.

Kearnes (1988) and Hilton (2006, p.313) identify the contractual interpretation of the functions of the state and of society itself as evidence of the influence of utilitarianism on the development of new approaches to public affairs. From this perspective, it can be understood that the best social results are obtained when individuals are left to pursue their respective interests, freely entering in contracts with one another in order to maximize their own happiness. The role of the government is that of arbiter and guardian, ensuring that these spontaneous contracts are observed correctly and intervening only when there is evidence that the pacts are malfunctioning in some way.

From such a standpoint, it could be claimed that the abject conditions of hygiene in the cities were proof that these contracts were not bringing about the intended well-being. A subject’s inaction or pursuit of private agendas could be seen as an interference in other people’s freedoms if it caused entire communities to be reduced to such a deplorable state that others were prevented from maximizing their own happiness. In more concrete terms, the poor provision of health and sanitation services by local entities or companies was indicative of a breach of the contract with taxpayers.

With the growing use of royal commissions came a more robust centralized bureaucracy. While the move to find legislative answers to social problems may not have been an invention of the 1830s, the idea of seeking solutions on a national scale was new if compared to the previous period (Hilton, 2006, p.313). Chadwick in particular gained a reputation for his extremely centralizing tendencies – a feature that caused friction throughout his public career. The report on pauperism was littered with portrayals of local authorities as corrupt and incompetent, and a similar tone was taken by other commissions. However, despite the creation of a central authority in the form of the Poor Law Commission, supported by legislation that made it compulsory for parishes to observe the guidelines of the reform, the commissioners’ text reveals a concern to keeping property owners engaged with the local administration, designing mechanisms that ensured they had control over the election of parish union councils. Underlying this approach was an awareness that any desire to introduce a standardized approach to local government would be impossible without the support of the ruling classes.^13^

Sanitation never acquired the same political capital as the new Poor Law, which meant any proposed interventions had to be presented with care. In their final report, the members of the Royal Commission on the Health of Towns went to some lengths to show how a move towards greater intervention in health matters by the national government would not be unprecedented. Before presenting their recommendations, they offer a historical overview of the laws pertaining to the subject, concluding that at least since the Magna Carta (1215), control over waterways and, consequently, the kingdom’s sewers had been under the jurisdiction of the Crown. In the ensuing centuries, the appointment of the first officials charged with undertaking sanitation works had been a royal prerogative. During the reign of Henry VIII, it had also been the king who had granted authorization for local entities to collect taxes to finance the opening of sewers. Based on these facts, the authors argue, the English legislative tradition supported the thesis that responsibility for the maintenance of good sanitary standards was not restricted to the private or local realm, but was also a matter of public and national scope (Great Britain, 1845, v.1, p.12-18).

Having established that its position has a centuries-long legal precedent, the commission forewarns readers that its proposals are not intended to supplant local powers. On the contrary, the powers of municipal or parish councils should be strengthened so that they can enforce the health agenda (Great Britain, 1845, v.1, p.23-25). This could be done by introducing centralization within the local area itself, reducing the overlapping of powers. Since 1725, infrastructure works and hygiene services in corporations had been instituted by private acts of Parliament, giving the local stakeholders responsible for executing the tasks the right to vote on such matters as road cleaning, sewer building, drainage, paving, lighting, and surveillance. Census data on property ownership were the norm for determining who had the right to vote on these matters or run for these positions, and often set the bar higher than was usual when determining who could run for parliamentary or municipal election. Popularly known as “improvement acts,” these laws created narrowly delineated jurisdictions, focusing on clearly defined territories and activities. Due to its statutory nature, this type of legislation remained in force even where local health councils were created in the wake of the 1848 act (McLean, 2006, p.33-34).

Efforts to introduce and organize health services in the manner envisaged by the commissioners were hindered by fragmented and overlapping patchworks of powers. This made it easier for local property owners to block any measures they deemed inconvenient. It also hampered the coordination of initiatives when issues that should be coordinated in harmony were split across different authorities. The Royal Commission on the Health of Towns gives an example of districts where the building of public roads and drainage channels was governed by different statutes and officials, when it was obvious that the two tasks were intertwined (Great Britain, 1845, v.1, p.65).

In view of the above, it is clear that the centralization of sanitation services was anything but the simple imposition of the national over the local. Much emphasis was placed on reducing the overlapping of responsibilities across parishes and towns. The proposed solution was to put control of the entire sanitation infrastructure in the hands of those responsible for sewage channels, whom Chadwick regarded as the most technical and skilled of the commissioners appointed under the Improvement Acts (Chadwick, 1842, p.309-316). The role of the Crown would be to appoint sanitary inspectors, when requested by local administrators or residents, who would then investigate the progress of the reforms and have powers to force local authorities to abide by the rules (Great Britain, 1845, v.1, p.40). This arrangement would not do away with the ability of property owners to influence the composition of the local councils, as no guidance is given on changing the criteria for the election of these groups. It was understood that having the support of the local elites was essential, not only politically, but also financially, as they were the ones responsible for levying taxes locally and ultimately for financing the works needed to adapt the urban infrastructure to the new standards.

In practice, Chadwick’s conception of sanitation saw engineering as more important than medical professionals in the development of disease prevention systems. This rationale was associated with a general feeling, since the cholera epidemic, that the medical community’s capacity to respond to public health crises was limited, making it more prudent to focus on preventive, environmental strategies. This spatial understanding of public health was not a consensus in the scientific community, even while it enjoyed greater official support. Paradigms that envisaged interfering in and hospitalizing bodies were not incompatible with the miasma theory and actually developed in parallel to it, as illustrated by the multiplication of hospitals financed by voluntary donations for people with fever and other illnesses, including cholera (Pickstone, 1999).

Whether through taxation or through charity, sanitary improvement measures required the support of property owners. Economic language is used in the commissions’ reports to highlight the benefits of the works. They stress the financial benefits to be assured by the improvements, making the provision of services cheaper through greater efficiency and avoiding future emergency expenditures. These investments, it is noted, would produce a healthier workforce, the advantages of which would not be limited to greater productivity and fewer working days lost to illness. Improved sanitation might remedy the illnesses of the body, but it would also help purge the vices that weakened the sense of decency and autonomy in the working classes, helping to diminish the levels of pauperism (Great Britain, 1844, v.1, p.10-11). These arguments were designed to overcome any resistance property owners might have to the intended works, which were considered costly and potentially disruptive of business activities, generating an apathy that frustrated the commissioners:

One officer, when asked how it was that in a district where fever had been rife nothing had been done under the authority of the law, which authorized its being cleansed? replied, that the Board had made precisely the same objections that were made when the cholera appeared; when it was proposed to cleanse the district, the answer made at the Board was, that ‘they did not believe it would do any good:’ and those of the officers who were landlords of the weekly tenements said, ‘Why should we disturb and drive away our tenants?’ and those who were shopkeepers said, ‘Why should we frighten away our customers by representing the neighbourhood as unhealthy?’ consequently nothing was done (Chadwick, 1842, p.322).

Interventions that might have an impact on property or economic relations were handled with care, and had to be negotiated and pared down to the bare essentials. Attachment to the miasma theory was one area that affected this. According to Kearns (1988), while miasmas encapsulated, in an extremely sensorial way, the idea of a state of socio-environmental imbalance in need of remedy, there were also more pragmatic reasons for preferring this approach. Admitting the existence of contagion would fuel calls for the quarantining of vessels in ports and isolation of infected areas, both of which would disrupt trade (Ringen, 1979; Baldwin, 2006, p.65). In its 1848 report, the commission of inquiry into health in the Metropolis spoke highly of the fact that such actions – pursued to address the first cholera epidemic – had been abandoned. The commissioners felt that belief in contagion caused governments to take overly coercive measures designed to interrupt the flow of people, which had already been proved ineffective and also spread panic among the population (Great Britain, 1848b, p.9). Embracing miasmas was a way of protecting the circulation of goods from disruption in the name of public health.

One upshot of the contractual conception of society and the spatial approach to public health was that labor relations remained largely outside the scope of the sanitary commissions. At a time when working conditions in factories were the target of successive inquiries, the commissioners reproduced an argument used by industrialists, claiming that health problems identified as typical of workers were caused by the urban environment, not their particular occupation. The contrast between the robust physical state and appearance of young people in the countryside and those who grew up in cities was held to indicate that working conditions were only marginally responsible for the health of workers, with housing and neighborhood condition being largely to blame (Chadwick, 1842, p.119).

The commissions that scrutinized occupational health most were the ones appointed to investigate the employment of children and youth, initially only in the cotton and textile factories, later expanding their scope to other activities. Intervening in the relationship between employer and employee was something that faced considerable resistance from business owners. Any regulation, they argued, might impair their profit margins, which were already squeezed by competition. As for the commissioners, they were unwilling to arbitrate what were perceived as agreements reached spontaneously between the parties, as this would set a dangerous precedent that would encourage organized workers and politicians hostile to the factory system to press for new labor legislation. The concessions made in this respect focused primarily on the use of child and youth labor, using as one of their justifications the fact that younger people were not autonomous subjects and were therefore incapable of entering into contracts (Great Britain, 1833, p.33-37, 52).^14^

Services that, by their very nature, were provided through local monopolies, as was the case of water, were an exception in the realm of market ethics, as they were not subject to competitive pressures on the same terms as other goods. Even in these cases, the commissions’ recommendations revolved around mitigating conflicts with the private sector. They suggested that local authorities acquire facilities from companies willing to sell them, or that public administration play the role of mediator between suppliers and consumers, using taxes to pay a fixed amount for the constant supply of water and preventing it from being denied to the poorest residents (Great Britain, 1833, p.77, 95-101).

Despite Chadwick’s talent for centralization, his ideas on sanitation were not presented in the commission reports as being in any way detrimental to local powers or in contradiction to property ownership and private interest. Balancing these principles was challenging, as shown by the failure of the Public Health Act and the General Board of Health, which can largely be explained by a lack of collaboration on the part of local property owners and authorities. This failure does not prevent us from recognizing the alignments between sanitary reform, on the one hand, and the market perspective and tradition of local self-management, on the other.

## Moral concerns and socio-environmental factors in the production of healthy workers

According to the reformers, poor sanitary conditions were associated not only with the debilitated physiological state of the working people, but also their moral and intellectual impairment. Numerous reports from the time refer to the “physical and moral condition” of the poor as correlated characteristics. In this narrative, disease was an evil that inhabited the same dwellings as criminality, drunkenness, pauperism, ungodliness, and unruliness.

At first glance, it would be understandable to credit the pathologization of poverty to the legacy of Malthusian thinking, but this association cannot be generalized. Both the authors of the report that inspired the new Poor Law, Chadwick and Nassau Senior, were critical of Malthus’s work (Bastos, 2023, p.263-265). Neither considered population increase a problem in itself, attributing the imbalances observed to institutions that were impeding the free movement of labor, causing there to be a surplus workforce in certain regions and activities. Chadwick also believed that Malthusianism, by naturalizing high mortality and attributing all evils to demographic principles, hindered the sanitary campaign by discrediting the search for objective solutions.^15^ The shadow of Malthus’s predictions still loomed over the collective political imagination, but it was still possible to harbor negative views of the poor without defending his ideas.

The trope of poverty as a disease spreading through the social fabric was nothing new. However, factors including liberalism and the political economy, evangelicalism, and the public health campaign left perceptible marks on this approach. Hilton (1988) coined the expression “Age of Atonement” to refer to the years of the Industrial Revolution and the reform movement. The term refers to an evangelical sentiment common to different denominations that, together with the liberalism of the political economy, helped shape the hegemonic sense of individuality among Britons in the nineteenth century. This articulation then propagated the idea of facing life as an ordeal to be overcome by practicing the values of temperance and perseverance in the face of the challenges inherent to life in a self-regulated market economy.

This factor is essential for us to understand the moral and individualistic interpretation of poverty and the illnesses and vices attributed to this state. The commissioners’ reports are riddled with passages that describe in somber tones the profligacy encountered among the poor, which reduced them to a state of bestiality and prevented them from adequately performing their duties as workers or parents as they gave themselves over to vice and dependence on charity. In an excerpt taken from the report on the condition of the Manchester working classes, written by James Kay, an educator and doctor on the Poor Law Commission, it is clear how much individual conduct was idealized as a solution to social problems:

If we would really improve the condition of the lower classes – if we would give them better habits, as well as make them better workmen – we ought to endeavour to make them acquainted with the principles that must determine their condition in life. The poor ought to be taught, that they are in a great measure the architects of their own fortune; that what others can do for them is trifling indeed, compared with what they can do for themselves; that they are infinitely more interested in the preservation of the public tranquillity than any other class of society; that mechanical inventions and discoveries are always supremely advantageous to them; and that their real interests can only be effectually promoted by their displaying greater prudence and forethought (Kay, 1832, p.61-62).^16^

Improved sanitation was seen as necessary to convert this demoralized worker into his opposite, the “independent worker.” His attributes had been presented in the report on the reform of the Poor Laws, in which he was contrasted with another archetype coined by the commissioners, that of the “assisted poor” (Great Britain, 1834, p.28-29, 50). What set the two apart was the individual’s nature. With his foresight and devotion to labor, the independent worker refused to depend on parish care. His virtues are developed further in subsequent commissions. In the inquiries into labor issues, especially the ones investigating the situation of hand-loom weavers impoverished by competition from the textile factories, the good worker’s sense of independence is enlarged upon. It is not just a matter of not seeking parish relief: with intelligence and resignation, the independent worker comprehends how futile it would be to rail against the inexorable laws of the market and how counterproductive and immoral it would be to appeal to trade unions or demagogic politicians, who insist on interventions to artificially – and unsustainably – augment the value of the workforce (Great Britain, 1841).

The health of workers and the virtues embedded in the ideal of independence were interlinked. The deterioration of sanitary conditions went hand in hand with immorality and a loss of appreciation for autonomy, as testified by the doctor Thomas Southwood Smith, who headed the Health of Towns Commission. When asked whether unhygienic conditions were correlated with pauperism, he replied: “Yes; and the consequent burden on private individuals and public institutions is the least part of the evil. The great evil of this state of things is its tendency to break down the spirit of independence, and to reduce large classes of the people to the degradation and wretchedness of depending for their support on charity, and not on their own industry” (Great Britain, 1844, v.1, p.10). The moral and individualizing judgment on the causes of poverty, however, did not always coincide with recognition that environmental factors, external to the individual, also had an impact on their power to act.

One line of thinking that was not ignored by contemporaries was that if squalor goes hand in hand with poverty, the sanitation issue could ultimately be explained by economic inequality. Chadwick did not disregard the socio-environmental aspect of the problem, but he knew that this approach would face political resistance: it would be easier for him to advance his career and conduct reforms if he made the technical discourse of infrastructure intervention the crux of his strategy (Mantovani, Marques, 2020).

The reports were unable to conciliate these two interpretations without a degree of discursive ambivalence. This trait is more prominent in the investigation carried out by Chadwick, which has a more visceral style than that used in the reports of the commissions investigating sanitation and health in towns and London itself. The roles attributed to the working classes in the health tragedy alternate between those of executioner and victim. Sometimes they are blamed for the misfortune of their situation, their rejection of the simplest hygiene measure, and the spread of vice and ignorance within the heart of the family. For example, reports about the involvement of the poor with mutual aid societies – popular funds whose members made small regular payments in exchange for financial assistance in the event of unemployment, illness, or death – are highly derogatory. Extreme cases are recounted by Chadwick and the commissioners investigating health in towns to illustrate the dishonesty of the poor, who attempt to obtain compensation from these associations by underhand means. Stories include parents who refuse to seek treatment for their children or who even poison them, hoping to pocket the money they will receive to cover the medical or funeral expenses (Chadwick, 1843, p.65-67; Great Britain, 1844, p.188-189).

Elsewhere, the poor are depicted more as those who are most harmed by a social structure that puts them at a disadvantage, leaving them exposed to the neglect of public authorities and the exploitation of private initiative: unscrupulous developers who build rows of precarious, insalubrious houses just to profit from rising rents; landlords who go so far as to collect rainwater then sell it to their tenants instead of ensuring them an adequate water supply (Chadwick, 1842, p.64-65). The examples of abuses of the poor are numerous. But supreme indignation is reserved for the supplementary report on burial practices. Even in death, society’s poorest are at the mercy of profiteering, entrusting the funeral rites of their loved ones to individuals who would do whatever it takes to charge more. This same rampant money-grabbing behavior also drove the practice of digging shallow graves in private cemeteries within the urban perimeter, which was not only in breach of public health precepts but also showed a lack of respect for the deceased – problems for which Chadwick suggested creating national cemeteries in the countryside (Chadwick, 1843, p.55-57, 102-103).

Jones (2013, p.503-505) uses the idea of “demoralization” to refer to the dominant language in social debates in nineteenth-century Britain until the 1870s. By the end of the century, “degeneration” had taken its place as the hegemonic rhetoric. While the former essentially reduced poverty and vice to individual choice, the latter was based on the understanding that individuals’ material and moral degradation was linked to socio-environmental factors. The time periods outlined by Jones do not, however, prevent us from recognizing that an environmental dimension was already manifested in mid-century discourses. The change in approach taken by the entities combating drunkenness in the 1850s is one such example (Hilton, 2006, p.577, 664). Indeed, as argued here, elements exogenous to individuals were already considered in the early analyses of the sanitary reform, even if they shared space with moralistic interpretations of the subject.

This brings us back to the utilitarian conception of a society governed by contractual interactions. However much the independent worker was seen as the outcome of self-improvement and responsibility for his own decisions, environmental conditions must exist for this virtuous cycle to emerge in the first place. Given the inability of private interests to promote an urban setting conducive to this without State intervention, it was down to the State to step in to provide the necessary balance.

It is worth noting how much the proximity between property owners and employers, on the one hand, and workers, on the other, was valued in helping improve the workers’ lot. On several occasions, mention is made of landlords or employers who were mindful of their tenants’ or employees’ hygiene, or who set up spaces for leisure and physical activities, bath houses, schools, worship, and other amenities for the community. The praise expressed for such initiatives, notwithstanding its paternalistic overtones, is a reminder that the reform and sanitary movements encapsulated the desires of the upwardly mobile middle classes, eager to see the extremities of the relationship between work and capital reconciled. Illustrating this perspective, in his 1842 report Chadwick reproduces the account of an officer from the parish union of Bedford, in which he describes the benefits gleaned when employers erected decent housing for their workforce:

The man sees his wife and family more comfortable than formerly; he has a better cottage and garden: he is stimulated to industry, and as he rises in respectability of station, he becomes aware that he has a character to lose. Thus an important point is gained. Having acquired certain advantages, he is anxious to retain and improve them; he strives more to preserve his independence, and becomes a member of benefit, medical, and clothing societies; and frequently, besides this, lays up a certain sum, quarterly or half-yearly, in the savings’ bank. Almost always attendant upon these advantages, we find the man sending his children to be regularly instructed in a Sunday, and, where possible, in a day-school, and himself and family more constant in their attendance at some place of worship on the Lord’s-day (Chadwick, 1842, p.262).

The social malaise depicted in so many accounts from the 1830s and 1840s was related to apprehensions spawned by the perception that society was divided – a perception that would be alleviated during the height of the Victorian era, in the third quarter of the century. From a reformist perspective, remedying this meant reconciling antagonisms so that the “wealthy and intelligent classes,” as the urban elites were called by the Metropolitan Sanitary Commission (Great Britain, 1845, v.1, p.2), could hold out a hand to guide the masses towards material and spiritual salvation. Moral instruction, industrious discipline, and acceptance of the free market reality were elements that, within a social environment balanced by the occasional intervention of public authorities, would have the effect of “eradicating the germs of pauperism from the rising generation” (Kay, 1839, p.3-4).

## Final considerations

Although the efforts of the sanitation model that took shape in the 1840s were frustrated in several respects, not least in the creation of a standardized network of local health councils, their impact should not be underestimated. The obsession with chasing miasmas was based on a mistaken hypothesis, but it led public health professionals to tackle real problems, improving the sanitation infrastructure and raising the nation’s awareness of the subject. Although reformist practices involved pragmatic strategies designed with objective purposes, they were part of a movement in which an ideological framework can be traced.

The establishment and maintenance of a *laissez-faire* order implied contradictions that demanded public action in the name of alleviating tensions and social ills, calling into question the virtues of the model of liberalism associated with industrialization. Reforms were designed to intervene on behalf of this socioeconomic arrangement, enabling its integration into society, even if, to do so, certain corollaries of this same trend had to be curbed. No new social order is consolidated through preconceived plans that translate an ideology directly into practice, but rather along winding routes of constant adaptations and resignifications in response to phenomena from the social reality. In such a situation, any rigid distinction between the fields of theory and practice will lose touch with the ideological component that is revealed in the very act of carrying out reform and in the contradictions inherent to this process.

The sanitation movement was part of a broader rationale that envisaged a model of society in which the exercise of public administration and the relationship between individuals and classes occurred along more institutionalized, standardized, rational lines, making liberal ethics more functional in the midst of growing urbanization and industrial development. Within this movement, there was still room for traces of paternalism: property owners could still impose their will on the machinery of government through the control they exerted in local councils, and charity was still a welcome ally, including for funding hospitals, dispensaries, schools, and other amenities needed to advance the campaign to raise moral and health standards among the working classes. For the sanitarians in the royal commissions, it was down to the government to ensure that towns and cities had a sanitary infrastructure that complied with the basic sanitation guidelines. But conceiving of a state that actively promoted a public health system with universal care was a far cry from its agenda.

## NOTES

1 “Evangelical revival” is a term used in British historiography to refer to the religious, evangelical fervor that swept the British Isles and the Thirteen Colonies in the 1730s and 1740s. It was marked by a trans-denominational urge for salvation, a need for “rebirth” through personal conversion, and the encouragement of preaching. In England, this movement was an important driver of the renewed expansion of dissenting circles after a period of retraction that came with the restoration of the monarchy in 1660. The impacts of this revival were not restricted to practitioners of evangelical religions. Many Anglicans began to advocate acts of worship that were more similar to those of the evangelicals, and the desire for atonement and elevation of the nation’s moral standards had consequences for society as a whole. I use the term “evangelicalism” to refer to this tendency in broad terms, as this is the word commonly used in British historiography.

2 These bodies, which were the equivalent of town councils, were elected in the boroughs that had a royal charter recognizing their right to self-government.

3 The complete collection of parliamentary papers from the nineteenth century has been digitalized and can be accessed via ProQuest’s UK Parliamentary Papers platform. Access is paid. However, almost all of the royal commissions’ inquiries have been digitalized and can be accessed on platforms such as Google Books and archive.org.

4 Over the course of its existence, a royal commission might produce multiple reports on local or regional matters before submitting its main report to Parliament, providing an overview of the results of the work carried out in the area under investigation. Depending on the progress of the work, this report could mark the conclusions of the inquiry, containing the commissioners’ recommendations and guidelines on the subject, or it could present partial considerations and explain that a new report would come out when more progress had been made. As for the Poor Law Commission of 1834 to 1847, it had some features that distinguished it from the other commissions studied. While the others were strictly royal commissions of inquiry, created solely to investigate and give an opinion on a given subject, the Poor Law Commission was also given the power to oversee the reform of the Poor Laws, issuing annual reports on the state of pauperism and the reformed system. It is important not to confuse the Poor Law Commission with the Royal Commission of Enquiry, which was in operation between 1833 and 1834, appointed to inquire into the operation of the Poor Laws and suggest reforms to improve it.

5 An official council consulted by the monarch for decision-making purposes, it is formed of experienced politicians who have usually served in Parliament.

6 Free translation from Spanish.

7 The Whig and Tory parties derived from positions taken in the crises that had pitted Parliament against the Crown since the seventeenth century. Traditionally, the Whigs tended to be in favor of curbing royal powers and defending the authority and independence of Parliament in relation to the monarchy. They were more inclined towards religious tolerance and, in the nineteenth century, more aligned with the industrial and urban bourgeoisies. The Tories, for their part, emphasized the power of the throne and the Anglican Church, although they recognized that the terms established with the Glorious Revolution should be respected. In 1835, they were restructured to form the Conservative Party.

8 In 1601, towards the end of the reign of Elizabeth I, the more than two dozen statutes enacted during the Tudor dynasty concerning poverty and vagrancy were compiled into a cohesive code, known as the Poor Law. The legislation empowered parishes to levy local taxes and elect officers to provide assistance to the poor who required public relief, for which the parish authorities were responsible. For an introduction to the origins of this institution, see Slack (1990).

9 For more on the reformers’ mistaken interpretation of pauperism, see Boyer (1990). For more on the continuities and discontinuities in the enforcement of the Poor Laws after the reform, see, in addition to the latter, Englander (2013) and Green (2010).

10
Fraser (1984, p.89-94) goes so far as to consider the new Poor Law the embryo of the National Health Service.

11 The term by which the inquiry reports presented to Parliament were known, since they were bound in blue.

12 For more on the different training options and occupations in the field of medicine and how its regulations defined the limits of each health profession in the nineteenth century, see Kanikadan and Marques (2013).

13 The desire to keep property owners directly involved in the management of pauperism is reflected in the commissioners’ justification for opting to retain local taxes, while rejecting the proposal for a national Poor Law tax (Great Britain, 1834, p.99-100). On the ways in which the 1834 amendment act increased property owners’ control over the Poor Law, see Boyer (1990, p.200-201).

14 For appraisals of child labor during the Industrial Revolution in England, see Kirby (2003) and Humphries (2010). On the health status of children involved in factory labor, see Kirby (2013).

15 For more on the impact of Malthus’s ideas on the medical community, see Hamlim and Gallagher-Kamper (2000).

16 This passage from Kay’s 1832 report is taken from an article published in the liberal journal *The Edinburgh Review* by the Scottish economist Ramsey McCulloch.
