# Supplementary material for: Eradicating the germs of pauperism: the political economy of the British sanitary movement in the 1830s and 1840s
Source: Hist Cienc Saude Manguinhos. 2024 Dec 16;31:e2024063. doi: 10.1590/S0104-59702024000100063en (PMC11654725; doi:10.1590/S0104-59702024000100063en)
Supplement: Translation

# Translation: Erradicar o germe do pauperismo: a economia política do sanitarismo britânico nas décadas de 1830 e 1840

**Authors:** Daniel Schneider Bastos

**Affiliations:** i Pesquisador em estágio pós-doutoral, Programa de Pós-graduação em História/Universidade Federal Fluminense. Niterói – RJ – Brasil danielbastos@id.uff.br

**Keywords:** História do século XIX, Saúde pública, Desenvolvimento industrial, Capitalismo, Sanitarismo

## Abstract

O artigo aborda o sanitarismo britânico das décadas de 1830 e 1840, propondo analisar a ideologia que perpassa os esforços oficiais pela promoção da saúde pública. As fontes utilizadas correspondem, principalmente, aos inquéritos movidos pelas comissões reais referentes ao estado sanitário das cidades. A interpretação defendida é a de que os pressupostos ideológicos que condicionam essas investigações podem ser compreendidos como parte de economia política do sanitarismo, dentro da qual se observam tensões oriundas das contradições existentes entre uma perspectiva liberal e a necessidade de um papel mais interventor por parte do poder público.

Na história social, o período da Primeira Revolução Industrial, ocorrida na Grã-Bretanha aproximadamente entre as décadas de 1780 e 1840, é também conhecido como Era da Reforma ou do Melhoramento. O malogro do governo na condução da crise das Treze Colônias desencadeou uma onda de insatisfação em relação ao governo, considerado corrupto, dispendioso e ineficiente, levando diferentes grupos sociais a se unirem sob a bandeira de uma reforma parlamentar capaz de elevar a sensação de transparência e de responsividade do poder público ante as demandas da sociedade. A noção de Era da Reforma, contudo, abarca significados para além da questão política e parlamentar, dado que outros sentidos vieram a se somar aos impulsos reformistas. Podemos destacar um sentido institucional, referente à mobilização pelo melhoramento de serviços e instituições, bem como um sentido moral e religioso marcado pela ânsia de expiação dos vícios da vida pública e privada, consequência do reavivamento do evangelicalismo^1^ no século XVIII (Innes, 2004).

As décadas de 1830 e 1840 formam uma conjuntura particularmente importante na implementação de iniciativas reformistas. A reforma eleitoral de 1832, complementada pela reforma das corporações municipais^2^ de 1835, dinamizou os mecanismos políticos e trouxe um influxo de concepções liberais para o Estado. Foram décadas de debate social intenso, na medida em que os resultados humanos da industrialização e da urbanização se tornaram mais impactantes na opinião pública. Entre as pautas estava o sanitarismo, que instigou repetidas investigações oficiais, imbuídas de significados políticos mais complexos do que a suposta objetividade das medidas sanitárias revelam à primeira impressão.

Este artigo tem por objeto a dimensão ideológica do sanitarismo britânico nas décadas de 1830 e 1840, especificamente a vertente associada a Edwin Chadwick, notório reformador atuante em diferentes frentes institucionais. A análise desenvolvida privilegia, como fontes primárias, os relatórios das comissões reais pertinentes ao tema, publicados nas décadas de 1830 e 1840. O estudo se concentra em publicações apresentadas por três comissões reais: a comissão da Lei dos Pobres (atuante entre 1834 e 1847), abordando os suplementos relativos ao problema da febre, presentes em seu quarto relatório, de 1838, e o relatório sanitário assinado por Chadwick, publicado em 1842 e que recebeu um suplemento no ano seguinte; a comissão de saúde das cidades (1843 a 1848), com dois relatórios, publicados em 1844 e 1845; a comissão de saúde metropolitana (1847 a 1850), que apresentou dois relatórios em 1848. Os relatos de outras comissões reais, que não tinham por objeto diretamente o sanitarismo, mas cujos inquéritos incidiram sobre o tópico de alguma maneira, também foram incluídos na pesquisa. É o caso dos relatórios das comissões para investigar o trabalho de crianças em fábricas (1833), o funcionamento da Lei dos Pobres (1833 a 1834) e a condição dos tecelões (1837 a 1841). Todos esses documentos, que integram o *Parliamentary Papers*, podem ser consultados em cópias físicas ou digitais.^3^

O trabalho das comissões é discutido a partir do conteúdo presente nos relatórios principais^4^ entregues ao Parlamento. Os anexos, que somam em regra um número de páginas superior ao dos relatórios conclusivos, contando com descrições iniciais de cenários locais ou regionais, retornos de questionários, cartas de instruções aos membros da comissão e outros materiais, não foram consultados para esta pesquisa. Também não foram analisados arquivos de correspondências internas e papéis burocráticos emitidos pelo comissariado no desempenho de suas funções.

A inclusão dessas fontes iria contribuir para compreender aspectos das comissões reais e seus atores que não estão explícitos nos relatórios finais. Todavia, essa escolha tornaria inviável o intuito de trabalhar com múltiplas comissões, impossibilitando o olhar transversal proposto. Assumindo a existência de limitações dentro da metodologia, argumenta-se, em contraponto, que o objetivo deste artigo não passa por desenhar com precisão um quadro da situação sanitária da Grã-Bretanha ou do movimento sanitarista de maneira geral. Como apontado por McLean (2006, p.x-xi), a pretensão de reconstituir trajetórias dessa natureza atendo-se aos atos parlamentares e às instituições centrais, como as comissões reais, arrisca colocar em segundo plano a importância das iniciativas nas localidades, espaços nos quais o jogo político trazia consequências mais diretas para a população britânica em seu cotidiano, inclusive por ser da esfera local que provinham os recursos para financiar empreendimentos sanitários.

O que se propõe é delinear os paradigmas que perpassam os encaminhamentos desse conjunto de comissões, de modo a colocar em evidência o quanto a visão de mundo que embasava uma vertente do sanitarismo, a qual desfrutou de embalo político no recorte estudado, estava atrelada a um viés liberal. O discurso utilizado nos relatórios conclusivos entregues pelos comissários, que representavam a síntese do trabalho dos integrantes de cada comissão e eram elaborados com a preocupação de encaminhar agendas políticas, é uma janela privilegiada para analisar essa articulação a partir da posição dos próprios sanitaristas. Por meio desses relatos, é debatida a forma como o liberalismo e outras correntes associadas incidem sobre o entendimento de sanitarismo e saúde pública que orientou a ação dos reformadores, ainda que se reconheça que esses condicionantes ideológicos mantenham suas contradições com aspectos práticos, inerentes à materialidade das demandas trazidas pelo capitalismo industrial e a nova geografia urbana. Defende-se que, por meio dessas contradições, é possível captar os contornos de uma economia política que se manifesta na campanha sanitária, em estreita relação com outras iniciativas reformistas durante o recorte estudado.

## Uma trajetória do sanitarismo britânico até o ato de saúde pública de 1848

O sanitarismo está inserido em uma complexa teia de tensionamentos sociais, envolvendo o decaimento dos indicadores de saúde nos centros urbanos, as mobilizações por melhores condições de trabalho, o aparecimento do Cartismo como movimento de massas, os esforços para padronizar e centralizar a gestão do pauperismo por meio da Lei dos Pobres, um *momentum* administrativo de qualificação dos quadros burocráticos, entre outros fatores que constituem a conjuntura politicamente tensionada das décadas de 1830 e 1840. Reconhecendo esse cenário intrincado e os múltiplos encadeamentos formados entre os elementos mencionados, não devemos descartar a relevância de fatos de caráter mais episódico para o desenvolvimento da pauta sanitária.

O aparecimento da cólera em forma epidêmica na Inglaterra em outubro de 1831, por meio do porto de Sunderland, é determinante nesse aspecto. O Conselho Central de Saúde havia sido recriado em junho de 1831, com sua composição dominada por integrantes do Royal College of Physicians londrino. Tratava-se de um corpo de assessoramento do Conselho Privado^5^ convocado diante de emergências sanitárias. Sua fundação deu-se em 1805, quando o Conselho Privado nomeou especialistas para auxiliar na formulação de medidas para evitar que a febre amarela chegasse ao Reino Unido. A junta foi dissolvida em 1806, sem que um novo conselho fosse convocado até a iminência da chegada da cólera. Embora tenha permanecido ativo durante os meses seguintes à sua refundação, o grupo detinha pouco poder efetivo para além de fornecer orientações ao governo central. O empoderamento para implementar as medidas de combate à cólera foi concedido aos conselhos organizados em nível local, com jurisdições restritas à respectiva paróquia ou município. Desde o final do século XVIII, conselhos locais de saúde já estavam em atuação em algumas cidades. O número dessas instituições, que ganharam reconhecimento legislativo mais definido com o Ato de Saúde Pública de 1848, multiplicou-se em resposta à crise epidêmica de 1831 e 1832, com a fundação de cerca de 1.200 conselhos durante o referido biênio (Brockington, 1961).

Finda a epidemia, o Conselho Central de Saúde foi desfeito, em dezembro de 1832. A legislação que expandiu os poderes dos corpos locais para combater a doença expirou sem ter continuidade. Prevalecia a mentalidade de que medidas sanitárias interventivas eram de natureza emergencial, não devendo constituir uma política contínua focada na prevenção.

A debilidade das instituições diante da cólera não significa que a doença não tenha impactado a consciência sanitária dos britânicos. A epidemia evidenciou a deterioração das condições de saúde nos centros urbanos e pôs em destaque o caráter social dessa degradação. Observadores munidos de dados estatísticos constataram o quanto a cólera se mostrava mais letal nas vizinhanças habitadas pelos trabalhadores, quando comparada a sua passagem por bairros mais ricos. Como relatou o médico espanhol Mateo Seoane (1832, p.10), pioneiro do sanitarismo na Espanha e que fora enviado para a Inglaterra para colher informações sobre a doença após o início da epidemia:

É de se notar também que quase todos os enfermos que houve em Sunderland se manifestaram na parte da cidade onde vive a gente pobre, pois aqui como em todas as demais partes da Inglaterra a cólera tem prevalecido principalmente nas moradas da miséria, e apenas quando se estendeu muito foi vista acometendo as pessoas ricas. É curioso observar a predileção deste mal terrível por espaços porcos, reduzidos e mal ventilados, e os pobres que os habitam; já se havia observado em Varsóvia, Berlim, Hamburgo e outros lugares esse fato, e confirmou-se do modo mais patente na Inglaterra.^6^

Dado o contexto do início da década de 1830, marcado por agitações populares por reforma política, a ação combativa de sindicatos que apenas recentemente tinham deixado a ilegalidade e um gasto público destinado ao pauperismo bem maior do que no começo do século, era como se o aparecimento da cólera confirmasse os temores expressos pelas vozes que alardeavam sobre o perigoso decaimento da condição física e moral da classe trabalhadora. Com um misto de interesse, estranhamento e horror, governo, nobreza e classe média pareciam ter ganhado, de súbito, a consciência de que coexistiam, em seu próprio país, com uma massa trabalhadora cujos hábitos e condições de vida soavam tão estrangeiros quanto os das comunidades nativas nos confins do Império. Referindo-se ao momento da chegada da cólera, a comissão real responsável por inquirir sobre o estado sanitário da metrópole londrina publicou em seu relatório de 1848 que:

Naquele tempo não apenas nenhum conhecimento havia sido adquirido pela experiência sobre o verdadeiro caráter dessa doença, mas nada se sabia sobre a condição real das classes que se provaram ser suas primeiras e mais fáceis vítimas, nem sobre o estado das localidades em que elas viviam. A metrópole do pobre não tinha nada em comum com a metrópole do rico, e como o mapa de Londres não exibe traços das vielas e travessas do pobre, assim mesmo os nomes desses lugares teriam àquele tempo soado tão estranhos aos habitantes de nossas grandes vias e praças quanto os das ruas de um país estrangeiro (Great Britain, 1848a, p.6).

Embora autoridades médicas e sanitárias estivessem cientes de que as dezenas de milhares de óbitos causados pela epidemia representavam um total comparável ao número de vidas ceifadas por doenças como varíola, tifo, influenza, difteria, tuberculose e afecções mais comuns do aparelho digestivo, a cólera mostrou-se socialmente mais disruptiva. Não era uma mazela relativamente naturalizada como outros males, causando histeria social ao se manifestar. Podendo levar à morte em apenas 12 horas após o surgimento dos primeiros sintomas, por meio de evacuações violentas que atentavam contra o senso de pudor em voga, a cólera assombrava o imaginário britânico. A ineficiência da medicina em prover tratamento eficaz elevou o pânico associado à doença (Evans, 1999, p.159-173).

A epidemia de cólera foi concomitante à intensificação das mobilizações pela reforma parlamentar, finalmente instituída com o Ato de Reforma de 1832. O ímpeto reformista abriu caminho para o retorno do partido *Whig* ^7^ ao poder após décadas de hegemonia conservadora. Entre as prioridades iniciais do novo governo, estava uma reforma da Lei dos Pobres^8^ que reduzisse as despesas dos contribuintes com o pauperismo e combatesse o que era percebido como um estado de imoralidade popular, induzido pela facilidade com a qual os pobres, supostamente, obtinham o socorro paroquial. Desde o final do século XVIII, uma conjuntura envolvendo rápida expansão demográfica, inelasticidade de oferta de trabalho no meio rural e inflação provocada pelas guerras contra a França resultou no aumento acelerado dos impostos para administrar o pauperismo. A hostilidade dentro do ambiente político e intelectual contra a Lei dos Pobres cresceu, tendo na obra de Malthus seu mais conhecido expoente (Malthus, 1998). Os Motins de Swing de 1830, nos quais trabalhadores rurais empobrecidos se revoltaram e destruíram propriedades no interior do país, asseguraram o apoio da classe fundiária a um projeto de reforma que fizesse do sistema paroquial uma ferramenta disciplinadora.

Nomeou-se em 1833 uma comissão real de inquérito para investigar o modo como a Lei dos Pobres estava funcionando na Inglaterra e no País de Gales, e quais seriam os meios para aprimorá-la. O relatório final do grupo foi concluído no ano seguinte (Great Britain, 1834). Já em 1834, o Parlamento aprovou o ato de emenda que, com base nas orientações da comissão, instituiu aquela que ficou conhecida como nova Lei dos Pobres. Pela nova legislação, as paróquias inglesas e galesas foram agrupadas em uniões paroquiais, cada qual incumbida de financiar a construção e manutenção de ao menos uma *workhouse*. Qualquer ajuda ofertada aos pobres deveria ter como contrapartida a internação voluntária nesses centros, desenhados para proporcionar uma experiência desagradável. Essa organização era baseada no princípio de que a condição daqueles amparados pela Lei dos Pobres deveria ser pior do que aquela encontrada pelos indivíduos empregados nos piores postos de trabalho disponíveis no mercado, assegurando que apenas os verdadeiramente desesperados continuassem a procurar o poder público (Great Britain, 1834, p.127-150).

Mesmo tendo sido baseada em pressupostos distorcidos sobre a realidade social, a reforma foi eficaz em reduzir os dispêndios com o pauperismo.^9^ Contudo, contrariando o discurso que embalou sua criação, a nova Lei dos Pobres se mostraria com o tempo um marco importante no acesso da população trabalhadora aos serviços públicos de saúde, uma vez que as *workhouses* mantinham médicos em seu corpo de funcionários e enfermarias em suas instalações. No decorrer das três décadas seguintes, esses centros foram se tornando cada vez mais associados à prestação de serviços sanitários, com a distinção entre pobre assistido e paciente tornando-se menos distinta.^10^

Com a função de conduzir a implementação prática da legislação aprovada, o ato de emenda de 1834 empoderou uma comissão real, denominada comissão da Lei dos Pobres, para atuar como autoridade nacional para assuntos relativos ao sistema reformado, perante a qual as uniões precisavam se reportar. Sua função era garantir que as novas diretrizes para o pauperismo fossem adotadas pelas localidades com a maior homogeneidade possível. A história dessa comissão está diretamente atrelada aos rumos do sanitarismo britânico. Foi por meio da sua atuação que as investigações sobre o estado sanitário do país começaram a se dar de modo mais sistemático por parte do governo. Em seu quarto relatório anual, de 1838, os comissários incluíram dois suplementos referentes às causas da febre entre os trabalhadores na região metropolitana, assinados pelos médicos a serviço do órgão. Ambos os escritos apontavam para a necessidade de se combater os miasmas, supostos gases nocivos que, emanando de matéria putrefata, causavam o aparecimento das doenças.

O primeiro documento, redigido por Neil Arnott e James Kay, elenca seis medidas para extinguir as exalações miasmáticas da área urbana, as quais seriam reiteradas pelos corpos de inquérito subsequentes: construir canais de esgoto conectando casas e ruas, com pavimentação adequada; garantir o fornecimento abundante de água para limpeza das vias públicas e consumo doméstico; contratação de varredores de rua para lidar com a sujeira que não podia ser removida pela água; melhorar a circulação de ar por meio do alargamento das vias, abertura de praças e remoção estratégica de imóveis; afastar dos centros mais movimentados atividades e ambientes tidos como geradores de miasmas, tais quais cemitérios, matadouros, criações de gado, manufaturas de gases, entre outros; evitar as aglomerações em hospedarias baratas (Great Britain, 1838, p.103-129). Thomas Southwood Smith, autor do outro suplemento, também ressaltou a importância de se prevenir a produção e concentração de miasmas. Defendeu, ainda, que hospitais de cólera fossem criados para tratar os infectados, evitando que estes fossem encaminhados para *workhouses*, nas quais poderiam transmitir a doença para os demais internos (Great Britain, 1838, p.129-151).

Os relatos instigaram o secretário da comissão da Lei dos Pobres, Edwin Chadwick, a aprofundar-se na pauta sanitária. Chadwick havia sido um dos responsáveis pela redação do relatório que encaminhou a reforma de 1834. Frustrado por não ter sido apontado como parte dos três comissários principais empossados com a nova legislação, aproveitou-se de seus contatos com políticos importantes para persuadir o Parlamento a requisitar uma investigação nacional sobre as condições sanitárias da população. Seu desejo foi atendido em 1839, quando foi autorizado a afastar-se de suas atribuições de secretário para se dedicar ao projeto.

O resultado foi publicado em 1842, um *best-seller* que venderia mais de cem mil exemplares. Seu suplemento, relativo às práticas de enterro nas cidades, foi apresentado no ano seguinte. O impacto do relatório se dá dentro daquilo que historiadores chamaram de “era de comissões” ou “era dos *blue books*”^11^ quando se referem ao segundo quarto do século XIX, indicando o aumento do número de inquéritos sociais publicados e a atração que esses escritos exerceram sobre a opinião pública (Hilton, 2006, p.602-604; Briggs, 2014, p.238). Alguns inquéritos eram fruto de ação individual, realizados por sujeitos interessados em divulgar seu ponto de vista sobre assuntos que julgavam merecer maior atenção. O governo, por sua vez, podia se valer de comitês parlamentares na realização de investigações, mas passou a apelar, com frequência, para o emprego de comissões reais de inquérito. Indicadas pelo Home Office, estas últimas podiam ser compostas por quaisquer indivíduos que os ministros avaliassem como capazes de contribuir para o assunto em questão.

Historicamente, esses órgãos eram objeto de polêmica, dado que eram corpos autônomos ao Parlamento, com origem na prerrogativa real de nomear agentes para tarefas de levantamentos de dados. A opção por recorrer a essa ferramenta mais constantemente no século XIX estava associada à consolidação do entendimento de que os comissários estavam a serviço do governo, e não da Coroa, reduzindo a impressão de que as comissões representavam uma intromissão da autoridade monárquica. Mas a expansão do emprego dos comissários também refletia a procura por nomes mais qualificados para inquirir sobre tópicos importantes e que não tivessem sua atuação tão afetada pelas disputas políticas, em um momento no qual a dinâmica bipartidária no Parlamento e nas eleições locais se cristalizava (Lockwood, 1967).

Uma prosopografia do conjunto de comissários que atuaram em comissões reais com temas sociais nas décadas de 1830 e 1840 revela origens sociais relativamente heterogêneas. Os filhos da classe fundiária, sobretudo integrantes da *gentry*, têm grande representatividade, mas dividem espaço com homens advindos de famílias de mercadores internacionais, profissionais e de alguns industrialistas e comerciantes menores. As carreiras tradicionais do direito e da medicina^12^ são as mais constatadas entre os comissários, mas observou-se também uma diversidade de outras ocupações que apontavam para o avanço da especialização e profissionalização em diferentes campos, incluindo engenheiros, geólogos, químicos, paleontólogos e economistas, além de clérigos, militares e homens de negócios. Alguns comissários eram *self-made men* que ascenderam por empreendimentos ou prestígio profissional. Havia a preocupação, no apontamento das comissões, de balancear critérios de mérito e competência com a força da linhagem e da tradição. Incorporava-se, dessa forma, talentos e capacidades adequados para realizar inquéritos considerados mais científicos e qualificados, contudo, sem abdicar de capital político para legitimar a ação desses órgãos e facilitar a colaboração dos poderes locais (Bastos, 2023, p.99-161). O próprio Chadwick era advindo da pequena classe média, filho de um jornalista radical. Sua ascensão na vida pública devia-se à proximidade que manteve com o filósofo e jurista Jeremy Bentham, cuja filosofia utilitarista foi uma influência determinante entre os reformadores sociais.

Diante da repercussão gerada pelo relatório de Chadwick em 1842, uma comissão real de inquérito foi designada em 1843 para esmiuçar o estado sanitário das cidades na Inglaterra e em Gales, tarefa que perdurou até 1848 e gerou dois relatórios principais (Great Britain, 1844, 1845). Uma segunda comissão foi nomeada especificamente para contemplar a metrópole londrina em 1847, atuando efetivamente até o ano seguinte, também apresentando dois relatórios (Great Britain, 1848a, 1848b). Ambas as iniciativas ratificaram as posições antes apresentadas por Chadwick, que acompanhou de perto todo o trabalho da primeira e integrou o corpo de comissários da última. A coroação de sua figura e dessa vertente do sanitarismo como política oficial se deu com a aprovação do Ato de Saúde Pública de 1848, o qual instituiu o Conselho Geral de Saúde, com Chadwick entre os três nomes principais. No ano seguinte, ele assumiria a direção da recém-criada Comissão Metropolitana de Esgotos, tornando-o a maior autoridade sanitária da capital.

Seu apogeu foi breve. Dotado de poderes teoricamente extensos para intervir a partir dos conselhos locais, faltava ao Conselho Geral de Saúde a prerrogativa de instituir compulsoriamente essas juntas nas localidades. Queixas sobre falta de financiamento, recursos humanos e colaboração por parte de outras instituições públicas deram o tom da experiência à frente do órgão. Já os limites da teoria sanitária de Chadwick foram evidenciados pela decisão equivocada de despejar os canais de esgoto no Thames, agravando a difusão da cólera, doença que voltou a se manifestar de forma epidêmica na Inglaterra entre 1848 e 1849, entre 1853 e 1854 e em 1866. Atritos com poderes locais, querelas contra companhias de água (detentoras de poderoso *lobby* no Parlamento, onde tinham muitos acionistas) e o fracasso em prevenir o retorno da mesma doença que facilitou sua ascensão são fatores que explicam o rápido desprestígio de Chadwick, compulsoriamente aposentado da carreira pública em 1854. O Conselho Geral de Saúde foi dissolvido em 1858, com suas funções sendo absorvidas pelo Conselho Privado. A relação entre autoridade nacional e local e o nível de intervenção do poder público sobre assuntos privados permaneceriam objeto de discussão, conforme novas estratégias para o problema sanitário eram desenhadas.

Longos debates foram travados na historiografia acerca da natureza do reformismo, incluindo a campanha sanitária. Muito dessa argumentação esteve orientada pela oposição entre interpretações das reformas como medidas *ad hoc*, voltadas para a solução objetiva e setorizada dos diferentes males constatados, e escolas voltadas para leituras mais ideológicas, preocupadas em identificar os traços que inserem a ação dos reformadores dentro de uma agenda ampla, imbuída pelas expectativas políticas e econômicas dos grupos que se fortalecem com a Revolução Industrial. Autores críticos ao segundo grupo costumam denunciar seu caráter supostamente “conspiracionista”, que ignora as *nuances* das frentes reformistas ao reduzir o seu conjunto a um plano de readequação social orquestrado pela burguesia e seus associados (Fraser, 1984, p.xxii-xxx).

Este artigo propõe uma economia política do sanitarismo como forma de superar essa dicotomia. Pretende-se, com isso, dar conta de reconhecer o aspecto pragmático existente no esforço dos reformadores, sem abdicar de conceber que sua atuação convergia em direções que revelam pressupostos ideológicos entranhados nesse movimento. O exercício efetivo da reforma, ao se dar sobre questões materiais da vida cotidiana, envolve inevitável grau de pragmatismo, reflexo das especificidades dessas situações. As dissonâncias que se fazem entre os planos teórico e prático, contudo, proporcionam contradições nas quais se explicitam os contornos da ideologia que se tensiona ante os desafios impostos pela realidade social e sua gestão. Dois dilemas que se sobressaem no sanitarismo irão nortear o restante deste estudo. Primeiro, o conflito entre pulsões centralizadoras e interventivas diante da defesa do *laissez-faire*. Em seguida, o desafio de conciliar a face socioambiental da saúde pública com uma leitura moral e individualizante desse mesmo tema.

## Intervenção e centralização em uma sociedade de *laissez-faire*

Entre liberais e conservadores alinhados aos dois partidos hegemônicos, a defesa da propriedade privada e das relações capitalistas era consenso. Somando-se à tradição inglesa de reservar autonomia para paróquias e corporações na condução dos assuntos locais, geridos por cargos e conselhos controlados por proprietários ou seus favoritos, esse posicionamento podia representar um desafio à instituição de medidas interventivas. Tanto a economia política quanto o utilitarismo, filosofias que influenciaram o reformismo liberal, jamais apresentaram o liberalismo como sinônimo de absoluta não intervenção. Ainda assim, a linha que separava a regulação legítima da violação intolerável foi tema de permanente tensão entre proprietários e reformadores.

Kearnes (1988) e Hilton (2006, p.313) apontaram para a leitura contratual da sociedade e das funções do Estado como uma contribuição da filosofia utilitarista para pautar novas abordagens dos assuntos públicos. Compreende-se, nesses termos, que os melhores resultados sociais são obtidos quando os indivíduos são deixados em liberdade para perseguir os respectivos interesses, firmando acordos livres entre si na busca pela própria felicidade. Ao governo cabe o lugar de árbitro, o guardião que zela pela correta execução desses contratos espontaneamente estabelecidos, sendo justa a sua interferência onde se constata o mau funcionamento dos pactos.

Era possível alegar, nesses termos, que as abjetas condições de higiene nas cidades eram prova de que esses contratos não estavam promovendo o bem-estar esperado. Ao permitirem, por descuido ou para atender a agendas particulares, que comunidades inteiras fossem rebaixadas a um estado lamentável de saúde, os sujeitos interferiam sobre a liberdade alheia ao criarem ambientes impróprios para que os demais pudessem maximizar sua própria felicidade. Mais concretamente, a má prestação de serviços sanitários por companhias ou órgãos locais apontava para a violação do contrato com os contribuintes.

O emprego crescente de comissões reais contribuiu para expandir uma burocracia centralizada. Procurar respostas legislativas para problemas sociais não foi uma invenção da década de 1830, mas o modo como as soluções passam a ser pensadas em dimensão nacional é uma diferença em relação ao período anterior (Hilton, 2006, p.313). Chadwick, em especial, ganhou fama como alguém extremamente centralizador, característica que lhe rendeu atritos por onde passou na carreira pública. Retratar poderes locais como forças corruptas e incompetentes foi uma tônica do relatório sobre o pauperismo, repetida por outras comissões. Contudo, apesar da criação de uma autoridade central na forma da comissão da Lei dos Pobres, amparada pela legislação que tornou compulsória a adesão das paróquias às diretrizes da reforma implementada, transparece no texto dos comissários a preocupação em manter os proprietários engajados com a gestão local, desenhando mecanismos que asseguravam o controle dessa categoria sobre a eleição dos conselhos das uniões paroquiais. Existia a consciência de que qualquer ambição para padronizar a administração não seria alcançada sem o apoio dessas elites.^13^

A questão sanitária jamais contou com o mesmo capital político que embalou a adesão à nova Lei dos Pobres, reforçando a necessidade de se apresentar com cautela proposições interventivas. Em seu relatório final, entregue em 1845, os comissários incumbidos de inquirir sobre a saúde nas cidades buscam demonstrar como o argumento por maior ingerência do governo nacional sobre assuntos sanitários não era um invencionismo recente. Antes de exporem suas recomendações, os autores fazem uma reconstituição histórica das leis referentes ao tema para concluir que, ao menos desde a Carta Magna de 1215, o controle sobre caminhos de água e, consequentemente, dos esgotos do reino, estavam sob jurisdição da Coroa. Nos séculos seguintes, foi por meio de prerrogativa real que se nomearam os primeiros oficiais encarregados de conduzir obras de saneamento. Também pela autoridade do rei, durante o reinado de Henrique VIII, foi concedida a autorização para os entes locais arrecadarem impostos para financiar a abertura de esgotos. Com base nesse retrospecto, é argumentado que a tradição legislativa inglesa dava suporte à tese de que a manutenção de padrões sanitários não era atribuição restrita ao privado ou à localidade, mas também um assunto de âmbito público e nacional (Great Britain, 1845, v.1, p.12-18).

Após ressaltar que sua posição possuía raízes antigas no direito, a comissão antecipa que suas proposições não têm por intenção suplantar as jurisdições locais. Ao contrário, insiste-se que os poderes de conselhos municipais ou paroquiais devem ser expandidos para que possam impor a agenda sanitária (Great Britain, 1845, v.1, p.23-25). A sugestão dada é a de fomentar a centralização dentro da própria localidade, reduzindo o ruído entre atribuições. Desde 1725, nas corporações, obras de infraestrutura e serviços de higiene eram instituídos por atos privados do Parlamento, autorizando que fossem eleitos na comunidade em questão responsáveis por gerenciar a execução de tarefas como limpeza de vias públicas, abertura de esgotos, drenagem, pavimentação, iluminação ou vigilância. Critérios censitários com base na propriedade eram a norma para determinar aqueles que possuíam o direito de votar ou se candidatar para essas posições, definindo patamares que poderiam ser mais restritivos do que aqueles que regulavam a participação nas eleições parlamentares ou municipais. Popularmente conhecidos como “atos de melhoramento”, essas leis criavam jurisdições estreitamente delineadas, incidindo sobre territórios e atividades bem especificados. Por seu caráter estatutário, esse tipo de legislação permanecia em vigor inclusive onde fossem criados conselhos locais de saúde amparados pelo ato de 1848 (McLean, 2006, p.33-34).

Os esforços para implementar e organizar os serviços de saúde nos moldes desejados pelos comissários esbarravam em mosaicos de jurisprudências fragmentadas e sobrepostas. Esse emaranhado facilitava que proprietários locais impusessem entraves às medidas sanitárias que considerassem inconvenientes. Também era difícil coordenar iniciativas quando questões que deveriam ser manejadas em conjunto estavam divididas entre autoridades distintas. A comissão de saúde das cidades menciona o exemplo de distritos nos quais a construção de vias públicas e a implementação de canais de drenagem eram regidas por estatutos e oficiais diferentes, quando era óbvio que se tratava de tarefas entrelaçadas (Great Britain, 1845, v.1, p.65).

Diante dessa situação, a centralização dos serviços sanitários não é apresentada como simples imposição do nacional sobre o local. Muita ênfase é dada à importância de reduzir a sobreposição de atribuições internamente em paróquias e municípios, propondo-se que o controle de toda a rede de infraestrutura sanitária fique concentrada nas mãos dos responsáveis pelos canais de esgoto, considerados por Chadwick (1842, p.309-316) as figuras mais técnicas e capacitadas entre todos os comissários nomeados via atos de melhoramento. O papel da Coroa seria o de apontar inspetores sanitários, quando estes fossem demandados por gestores ou residentes da comunidade em questão, capazes de averiguar o andamento das reformas e com poderes para coagir as autoridades locais a se adequarem às normas (Great Britain, 1845, v.1, p.40). Esse arranjo não esvaziava a capacidade dos proprietários de influenciar a composição de conselhos locais, visto que nenhuma orientação é dada no sentido de alterar o caráter censitário da eleição desses grupos. Sabia-se que a adesão das elites locais era indispensável, não apenas em termos de apoio político, mas também financeiro, pois seria a classe proprietária que, via impostos diretos levantados localmente, iria arcar com os custos de adequação da malha urbana aos novos padrões.

A concepção de sanitarismo de Chadwick reservava, na prática, maior relevância à engenharia na elaboração de sistemas de prevenção do que aos profissionais da medicina. Tal lógica estava associada à sensação deixada pela cólera de que a comunidade médica tinha capacidade limitada para reagir às moléstias, sendo mais prudente apostar em estratégias preventivas de ação sobre o ambiente. Esse entendimento espacial da saúde pública não era consenso na comunidade científica, mesmo nos anos em que gozou de maior respaldo oficial. Paradigmas voltados para a intervenção sobre os corpos e sua internação não eram incompatíveis com a hipótese miasmática e se desenvolviam paralelamente, como ilustrado pela multiplicação de hospitais financiados por arrecadação voluntária para receber pessoas com febre e outros males, inclusive a cólera (Pickstone, 1999).

Seja pelos impostos, seja pela caridade, as iniciativas de melhoria sanitária demandavam o apoio dos proprietários. Uma linguagem econômica é usada nos relatórios das comissões para enaltecer os benefícios das obras. É enfatizado que os melhoramentos têm retorno financeiro assegurado, barateando a prestação dos serviços ao torná-los mais eficientes e evitando futuros custos emergenciais. Ressalta-se que esses investimentos produziam uma força de trabalho mais saudável. A vantagem não se resumiria à maior produtividade e a menos dias de labuta perdidos por adoecimento. O sanitarismo iria remediar as enfermidades do corpo, mas também ajudaria a expurgar os vícios que debilitavam o senso de decência e autonomia nas classes populares, contribuindo para reduzir as fileiras do pauperismo (Great Britain, 1844, v.1, p.10-11). Esse esforço de convencimento era uma tentativa de superar a resistência dos proprietários em relação às obras, consideradas dispendiosas e com potencial para atrapalhar os negócios, gerando uma apatia que frustrava o comissariado:

Um oficial, quando perguntado sobre como que em um distrito onde a febre estava abundante nada havia sido feito sob a autoridade da lei que autorizara sua limpeza, replicou que o conselho fizera precisamente as mesmas objeções que foram feitas quando a cólera apareceu; quando foi proposto limpar o distrito, a resposta dada ao conselho foi que ‘eles não acreditavam que faria bem algum’: e aqueles entre os oficiais que eram proprietários das terras dos imóveis de aluguel disseram, ‘Por que deveríamos perturbar e afastar nossos inquilinos?’ e aqueles que eram lojistas disseram, ‘Por que deveríamos afugentar nossos clientes ao apresentar nossa vizinhança como insalubre?’, consequentemente nada era feito (Chadwick, 1842, p.322).

Intervenções sobre o campo da propriedade e das relações econômicas são tratadas delicadamente, devendo ser feitas de forma negociada e reduzindo-se ao indispensável. O apego à teoria miasmática passa por essa questão. Conforme Kearns (1988), os miasmas sintetizavam de maneira extremamente sensorial a ideia de um estado de desequilíbrio socioambiental a ser remediado, mas existiam também razões de ordem mais pragmática para se preferir essa abordagem. Admitir a existência do contágio elevava o apelo pelas práticas de quarentena das embarcações nos portos e o isolamento de áreas infectadas, práticas disruptivas do ponto de vista comercial (Ringen, 1979; Baldwin, 2006, p.65). Em seu relatório de 1848, a comissão de inquérito para a saúde metropolitana celebra que essas ações, praticadas por ocasião da primeira epidemia de cólera, tenham sido abandonadas. Consideram que a crença no contágio incentiva o exercício de medidas demasiado coercitivas por parte dos governos, baseadas na interrupção do fluxo de pessoas, cuja ineficácia já estava comprovada e que disseminavam o pânico na população (Great Britain, 1848b, p.9). Abraçar os miasmas era uma forma de resguardar a circulação de mercadorias de interdições em nome do sanitarismo.

Uma consequência da concepção contratual da sociedade e da abordagem espacial da saúde pública foi o fato de que as relações de trabalho permaneceram relativamente fora do escopo das comissões sanitárias. Em um momento no qual as condições de trabalho nas fábricas eram alvo de sucessivos inquéritos, os comissários reproduzem um argumento utilizado pelos industrialistas, alegando que problemas de saúde apontados como típicos do operariado eram causados pelo ambiente urbano, mas não pela ocupação em particular. O contraste entre a desenvoltura física e o aspecto dos jovens do campo, quando comparados àqueles que cresceram nas cidades, seria um indicativo de que os empregos tinham uma parcela pequena de responsabilidade sobre a saúde dos trabalhadores, sendo muito mais determinante o estado das residências e vizinhanças nas quais habitavam (Chadwick, 1842, p.119).

As comissões que mais se aprofundaram na saúde do trabalho foram aquelas nomeadas para investigar o emprego de crianças e jovens, inicialmente apenas nas fábricas de tecidos de algodão, posteriormente ampliando seu escopo para outras atividades. Intervir sobre os vínculos entre os empregadores e seus funcionários era algo que enfrentava grande resistência da classe patronal. Qualquer regulação, argumentam os patrões, poderia comprometer margens de lucro já muito deprimidas pela competição. Por parte dos comissários, havia indisposição para arbitrar o que era percebido como acordos espontaneamente estabelecidos entre as partes, abrindo um precedente perigoso para trabalhadores organizados e políticos hostis ao sistema fabril pressionarem por novas legislações trabalhistas. As concessões feitas nesse aspecto incidiram sobretudo sobre o uso de mão de obra infantil e juvenil, usando-se como uma de suas justificativas o fato de que os mais novos não eram sujeitos autônomos, e, portanto, estavam inaptos para firmar contratos (Great Britain, 1833, p.33-37, 52).^14^

Serviços que, por natureza, eram prestados por meio de monopólios locais, como era o caso do fornecimento de água, ocupavam posição excepcional na ética de mercado, pois não estavam sujeitos à pressão competitiva nos mesmos termos que outras mercadorias. Mesmo nesses casos, as recomendações passam por atenuar conflitos com o setor privado. Sugerem que os poderes locais façam a aquisição das instalações de companhias dispostas a vendê-las, ou que a administração pública faça o papel de mediadora entre fornecedores e consumidores, utilizando impostos para pagar uma quantia fixa pelo suprimento constante de água, evitando que seu fluxo seja vedado aos residentes mais pobres (Great Britain, 1833, p.77, 95-101).

Não obstante o gênio centralizador de Chadwick, seu sanitarismo não foi apresentado nas comissões como um movimento em detrimento dos poderes locais, ou que mostrasse contradições com o viés da propriedade e do interesse privado. Equilibrar esses princípios era desafiador, como mostrou o fracasso da experiência do Ato de Saúde Pública e do Conselho Geral de Saúde, em muito explicado pela falta de colaboração por parte dos proprietários e autoridades nas localidades. Esse insucesso não nos impede de reconhecer os alinhamentos da reforma sanitária com a perspectiva de mercado e a tradição de autogestão local.

## O problema da moral e dos fatores socioambientais na produção de trabalhadores sadios

A degradação sanitária, para os reformadores, não implicava apenas o decaimento das condições fisiológicas da população trabalhadora, mas igualmente seu enfraquecimento moral e intelectual. Numerosos relatos da época fazem referência à “condição física e moral” dos pobres como características correlatas. Nessa narrativa, a doença era um mal que habitava as mesmas moradas do crime, do alcoolismo, do pauperismo, da irreligião e da insubordinação.

É um impulso compreensível creditar a patologização da pobreza ao legado do pensamento malthusiano, mas essa associação não pode ser generalizada. Ambos os redatores do relatório que inspirou a nova Lei dos Pobres, Chadwick e Nassau Senior, eram críticos à obra de Malthus (Bastos, 2023, p.263-265). Nenhum dos dois considerava o aumento populacional um problema em si, atribuindo os desequilíbrios observados às instituições que estariam impedindo a livre circulação de mão de obra, produzindo trabalho excedente em determinadas regiões e atividades. Chadwick julgava ainda que o malthusianismo, por naturalizar a alta mortalidade e atribuir todos os males ao princípio demográfico, atrapalhava a campanha sanitária por desacreditar a busca por soluções objetivas.^15^ A sombra das previsões de Malthus ainda se projetava sobre o imaginário político, mas era possível nutrir opiniões negativas sobre os pobres sem aderir às ideias do economista.

A alegoria da pobreza como uma doença espalhando-se pelo tecido social não era novidade. Todavia, fatores que incluem o liberalismo e a economia política, o evangelicalismo e a campanha pela saúde pública imprimiram marcas perceptíveis nessa abordagem. Hilton (1988) cunhou a expressão “Era de Expiação” para se referir ao período compreendendo a Revolução Industrial e o reformismo. O termo coloca em evidência um sentimento evangelicalista transversal às denominações que, ao lado do liberalismo da economia política, ajudou a moldar o senso de individualidade hegemônico dos britânicos no século XIX. Essa articulação disseminou a perspectiva de encarar a vida como uma provação a ser vencida pela exibição de valores de temperança e esforço obstinado perante os desafios inerentes à vida em uma economia de mercado autorregulado.

Esse fator é indispensável para compreender a leitura moralista e individualista da pobreza e das doenças e vícios atribuídos a esse estado. Os relatórios dos comissários são permeados de passagens que descrevem em cores soturnas o grau de imoralidade constatado entre os pobres, reduzidos a um estado de bestialização que os impedia de exercer adequadamente suas funções como trabalhadores ou os cuidados parentais, entregando-se ao vício e à dependência da caridade. No trecho retirado do relato sobre a condição do operariado de Manchester de autoria de James Kay (1832, p.61-62), médico da comissão da Lei dos Pobres e educador, fica evidente como a conduta individual era idealizada como solução para problemas sociais:

Se vamos melhorar realmente a condição das classes mais baixas – se iremos dar a eles hábitos melhores, assim como fazer deles melhores trabalhadores –, devemos nos esforçar para fazê-los familiarizados com os princípios que devem determinar sua condição na vida. Os pobres devem ser ensinados que eles são em grande medida arquitetos de sua própria fortuna; que o que os outros podem fazer por eles é insignificante, comparado com o que eles podem fazer por si mesmos; que eles são infinitamente mais interessados na preservação da tranquilidade pública do que qualquer outra classe da sociedade; as invenções e descobertas mecânicas são sempre supremamente vantajosas para eles; e que seus interesses reais podem apenas ser efetivamente promovidos ao demonstrarem grande prudência e previdência.^16^

O sanitarismo era tido como necessário para converter esse trabalhador desmoralizado em seu oposto, o “trabalhador independente”. Seus atributos haviam sido apresentados no relatório sobre a reforma da Lei dos Pobres, no qual é contraposto a outro arquétipo cunhado pelo comissariado, o do pobre assistido (Great Britain, 1834, p.28-29, 50). O caráter diferenciava ambos. Por seu senso de previdência e dedicação à labuta, o trabalhador independente se recusava a entregar-se aos cuidados paroquiais. Suas virtudes são desenvolvidas em comissões subsequentes. Nos inquéritos que se debruçaram sobre temas trabalhistas, sobretudo aqueles que investigaram a situação dos tecelões empobrecidos pela competição com a tecelagem fabril, o sentimento de independência que caracteriza o bom trabalhador é descrito de forma expandida. Não se trata, apenas, de manter-se longe do socorro dos paroquianos. O trabalhador independente é aquele que, com inteligência e resignação, mostra-se consciente da inutilidade de se debater contra leis inexoráveis de mercado, sabendo ser contraproducente e imoral apelar para sindicatos ou políticos demagógicos, que insistem em intervenções para elevar de modo artificial e insustentável o valor da força de trabalho (Great Britain, 1841).

A saúde do trabalhador e as virtudes embutidas no ideal de independência estavam mutuamente atreladas. A deterioração das condições sanitárias caminhava de mãos dadas com a imoralidade e a perda de apreço pela autonomia. Conforme o testemunho do médico e comissário real Thomas Southwood Smith diante da comissão para a saúde das cidades, ao ser indagado se a falta de higiene mantinha correlação com o pauperismo: “Sim; e o consequente fardo sobre indivíduos privados e instituições públicas é a menor parte desse mal. O grande mal desse estado de coisas é a tendência a quebrar o espírito de independência, e a reduzir amplas classes do povo à degradação e perversidade de dependerem para seu suporte da caridade, e não de sua própria indústria” (Great Britain, 1844, v.1, p.10). O juízo moral e individualizante sobre as causas da pobreza, todavia, possuía contradições com o reconhecimento de que fatores ambientais, externos ao indivíduo, incidiam sobre as possibilidades do último.

Um raciocínio que não era ignorado pelos contemporâneos era o de que, se a imundície caminha ao lado da pobreza, o drama sanitário era explicado, em última instância, pela desigualdade econômica. Chadwick não desprezava a face socioambiental do problema, mas sabia que essa abordagem enfrentaria resistência política, sendo mais fácil emplacar sua carreira e conduzir reformas reivindicando o discurso técnico de intervenção sobre a infraestrutura como base de sua estratégia (Mantovani, Marques, 2020).

A equalização dessas duas leituras não se deu sem que certa ambivalência discursiva transparecesse nos relatórios. Esse traço é mais saliente na investigação feita por Chadwick, dotada de estilo mais visceral do que aquele usado nos relatórios das comissões para a saúde das cidades provincianas e da zona metropolitana. A posição atribuída às classes trabalhadoras na tragédia sanitária alterna entre os papéis de algoz e de vítima. Ora são responsabilizadas pelo infortúnio de sua situação, rejeitando os hábitos higiênicos mais simples e fomentando o vício e a ignorância no seio familiar. São fortemente depreciativos, por exemplo, os relatos sobre o envolvimento dos pobres com as sociedades de ajuda mútua, fundos populares que, em troca de um pequeno pagamento regular, ofereciam aos membros ajuda financeira em caso de desemprego, adoecimento ou óbito. Episódios pitorescos foram reunidos por Chadwick e pela comissão de saúde das cidades para ilustrar a desonestidade dos pobres, que tentam obter compensação dessas associações por meios ardilosos. As histórias incluem pais que se recusam a buscar tratamento para os filhos ou que chegam a envenená-los, na expectativa de embolsar o dinheiro pago como auxílio para despesas médicas ou funerárias (Chadwick, 1843, p.65-67; Great Britain, 1844, p.188-189).

Em outros momentos, o quadro se inclina de modo a retratá-los como os mais prejudicados por uma estrutura social que os desfavorece, na qual estão expostos ao descaso do poder público e à exploração da iniciativa privada. Empreiteiros inescrupulosos, que erguem fileiras de moradias precárias e insalubres apenas para lucrar com a alta dos aluguéis nas cidades. Proprietários mesquinhos a ponto de reter a água da chuva para vender aos seus inquilinos, em vez de assegurar o seu fornecimento adequado (Chadwick, 1842, p.64-65). Numerosos eram os exemplos de práticas abusivas às quais os pobres estavam submetidos. O máximo da indignação ficou reservado para o volume suplementar, dedicado às práticas de enterro. Mesmo na morte, os mais humildes estavam expostos à sanha por lucro, confiando os ritos fúnebres de seus entes queridos a agentes que fariam todo o possível para encarecer os procedimentos. Essa mesma sede desgovernada por dinheiro motivava a abertura de covas rasas em cemitérios privados, escavados às pressas dentro do perímetro urbano, um atentado à saúde pública e desrespeito pelos finados, problemas para os quais Chadwick sugere a criação de cemitérios nacionais no interior (Chadwick, 1843, p.55-57, 102-103).

Jones (2013, p.503-505) utilizou a ideia de “desmoralização” para se referir à linguagem dominante nos debates sociais do século XIX britânico até a década de 1870. No final do século, a “degeneração” assumiria o lugar como retórica hegemônica. Enquanto a primeira reduzia a miséria e o vício, essencialmente, à escolha individual, a última se baseava no entendimento de que a degradação material e moral dos sujeitos estava atrelada aos fatores socioambientais. A periodização esboçada por Jones, contudo, não nos impede de reconhecer que uma dimensão ambiental já se manifestava em discursos de meados do século. A mudança de abordagem das entidades de combate ao alcoolismo na década de 1850 é um exemplo nessa direção (Hilton, 2006, p.577, 664), e, como argumentado aqui, elementos exógenos aos indivíduos já tinham lugar relevante nas análises do início da reforma sanitária, mesmo que dividindo espaço com a leitura moralista do assunto.

Retornamos à concepção utilitarista de uma sociedade regida por interações contratuais. Por mais que o trabalhador independente fosse enxergado como fruto do autocultivo e da responsabilidade por suas decisões, condições ambientais deveriam existir para que esse ciclo virtuoso pudesse aflorar em primeiro lugar. Diante da incapacidade do interesse privado em promover, sozinho, um espaço urbano nessas condições, era dever do Estado entrar em ação para propiciar o equilíbrio necessário.

Cabe observar o quanto a aproximação entre proprietários e empregadores, de um lado, e trabalhadores, do outro, era valorizada como contribuição para elevar a condição dos últimos. Casos de proprietários ou patrões que mantêm o olhar atento sobre os hábitos higiênicos de seus inquilinos ou empregados, ou que instituem espaços para lazer, atividades físicas, casas de banho, escolas, culto e outras benesses acessíveis para a comunidade são mencionados em diversas ocasiões. A exaltação dessas atitudes, imbuídas de aroma paternalista, são um lembrete de que o reformismo e o sanitarismo expressavam os anseios da classe média ascendente, desejosa de ver conciliadas as extremidades da relação entre trabalho e capital. Ilustrando essa perspectiva, Chadwick reproduziu em seu relatório de 1842 o relato de uma autoridade da união paroquial de Bedford, na qual são descritos os benefícios oriundos da prática de alguns empregadores de erguer casas adequadas para sua força de trabalho:

O homem vê sua esposa e família mais confortáveis do que antes; ele tem um *cottage* ^17^ e jardim melhores; ele é estimulado para a industriosidade, e conforme ele ascende em respeitabilidade se torna consciente de que tem um caráter a perder. Assim um objetivo importante é alcançado. Tendo adquirido certos benefícios, ele anseia por retê-los e melhorá-los; ele se esforça mais para manter sua independência e se torna um membro de sociedades de ajuda, médicas e de roupas; e frequentemente, além disso, deposita uma certa soma, trimestral ou semestralmente, no banco de poupança. Quase sempre concomitante a essas vantagens, encontramos o homem enviando seu filho para ser regularmente instruído em uma escola dominical, e, onde possível, em uma escola diária, e ele mesmo e sua família, mais constantes em algum local de culto no Dia do Senhor (Chadwick, 1842, p.262).

O mal-estar social registrado em tantas impressões das décadas de 1830 e 1840 está relacionado à apreensão diante de uma sociedade que parece cindida, sensação que seria atenuada pelo apogeu do vitorianismo no terceiro quarto do século. Remediar essa situação passava, na visão reformista, por conciliar antagonismos de modo que as “classes ricas e inteligentes”, como foram chamadas as elites urbanas pela comissão de saúde metropolitana (Great Britain, 1845, v.1, p.2), pudessem estender a mão para orientar as massas na direção da salvação material e espiritual. Instrução moral, disciplina de trabalho e aceitação da realidade de livre mercado eram elementos que, dentro de um ambiente social equilibrado pela pontual intervenção do poder público, iriam “erradicar os germes do pauperismo da geração em ascensão” (Kay, 1839, p.3-4).

## Considerações finais

Embora, em diversos aspectos, sobretudo na criação de uma rede padronizada de conselhos locais de saúde, os esforços do modelo de sanitarismo que tomou forma na década de 1840 tenham sido frustrados, seus efeitos não devem ser menosprezados. A obsessão em perseguir os miasmas estava pautada por uma hipótese equivocada, mas ela levou os sanitaristas a agir sobre problemas reais, aprimorando a infraestrutura sanitária e elevando a consciência nacional sobre o tema. Embora a prática reformista envolvesse estratégias de caráter pragmático, desenhadas com finalidades objetivas, é possível delinear um arcabouço ideológico em torno desse movimento.

O estabelecimento e a manutenção de uma ordem de *laissez-faire* implicavam contradições que demandavam ação pública, em nome de amenizar tensões e males sociais que colocavam em xeque as virtudes do modelo de liberalismo associado à industrialização. A atuação do reformismo se deu no sentido de intervir em nome desse arranjo socioeconômico, viabilizando seu entranhamento na sociedade mesmo que, para isso, fosse necessário coibir determinados desdobramentos dessa mesma tendência. A formação de uma nova ordem social não se consolida por meio de tramas pré-concebidas, ideologia que se traduz em prática por caminhos lineares, mas sim por etapas sinuosas de constante adaptação e ressignificação diante de fenômenos postos pela realidade social. Uma distinção rígida entre os campos da teoria e da prática, nessa situação, bota a perder precisamente o componente ideológico que se revela no ato de fazer reforma e nas contradições inerentes a esse processo.

O sanitarismo está inserido na lógica de implementação de um modelo de sociedade no qual o exercício da administração pública e a relação entre indivíduos e classes se dá em linhas mais institucionalizadas, padronizadas e racionais, tendo por horizonte tornar mais funcional a ética liberal em meio ao cenário de urbanização e desenvolvimento industrial. Esse projeto não excluía brechas para traços de paternalismo e pessoalidade: os proprietários poderiam continuar a impor suas feições na máquina governamental, pelo controle dos conselhos locais, e a caridade permanecer uma aliada bem-vinda, inclusive pelo financiamento de hospitais, dispensários, escolas e outros centros indispensáveis para fazer avançar a campanha de recuperação dos padrões morais e sanitários entre a classe trabalhadora. Para os sanitaristas nas comissões reais, era função do governo agir para que a infraestrutura sanitária das cidades obedecesse a critérios básicos de saneamento. Mas conceber um Estado que promovesse ativamente um sistema de saúde pública, dotado de serviços de atendimento e cuidado de caráter universal, era algo distante de sua pauta.

## NOTAS

1 A expressão *evangelical revival* (reavivamento evangélico) é usada na historiografia britânica para se referir ao ardor religioso de caráter evangélico que varreu as Ilhas Britânicas e as Treze Colônias nas décadas de 1730 e 1740. Caracterizou-se pelo sentimento transdenominacional de busca pela salvação, pela necessidade de “renascer” por meio da conversão pessoal e pelo incentivo à pregação. Na Inglaterra, esse movimento foi importante para que os círculos dissidentes voltassem a se expandir, após terem experimentado uma retração com a restauração da monarquia em 1660. Os impactos dessa retomada não ficaram restritos aos praticantes de religiões evangélicas. Muitos anglicanos passaram a defender experiências de culto mais próximas às práticas evangélicas, e o desejo de expiação e elevação dos padrões morais da nação trouxe consequências para a sociedade em sua totalidade. Optei pelo termo “evangelicalismo” para me referir a essa tendência em sua amplitude, tradução literal de *evangelicalism*, palavra costumeiramente usada na historiografia britânica com a mesma finalidade.

2 Corpos equivalentes às prefeituras, eleitos nos burgos que gozavam de carta régia reconhecendo seu direito de autogovernança.

3 A coleção completa dos *Parliamentary Papers* referentes ao século XIX encontra-se, atualmente, digitalizada e pode ser acessada pela plataforma UK Parliamentary Papers, da ProQuest. Trata-se de um acesso pago. Os inquéritos das Comissões Reais, contudo, podem ser quase todos encontrados digitalizados em plataformas como Google Books e archive.org.

4 Ao longo de sua existência, uma comissão real poderia produzir múltiplos relatórios com impressões locais ou regionais antes de entregar ao Parlamento o seu relatório principal, trazendo o panorama dos resultados do trabalho realizado em todo o território investigado. A depender do andamento do trabalho dos comissários, esse relatório poderia ser o documento conclusivo da investigação, trazendo os encaminhamentos e orientações do comissariado em relação ao tema, ou poderiam ser apresentadas considerações parciais juntamente com o anúncio de que um novo relatório seria emitido quando houvesse maiores avanços. Sobre a comissão da Lei dos Pobres de 1834 a 1847, esta possui algumas especificidades em relação às demais comissões estudadas. Enquanto as últimas eram estritamente comissões reais de inquérito, criadas apenas para investigar e dar um parecer sobre determinado assunto, a comissão da Lei dos Pobres foi instituída como um órgão com poderes ampliados para gerenciar a reforma da Lei dos Pobres, entregando relatórios anuais sobre o estado do pauperismo e do sistema reformado. É importante não confundir a comissão da Lei dos Pobres com a comissão real de inquérito que esteve em ação entre 1833 e 1834, nomeada para inquirir sobre o funcionamento da Lei dos Pobres e sugerir reformas para aprimorá-la.

5 Conselho oficial consultado pelo monarca para a tomada de decisões. É formado por políticos experientes, via de regra, com passagem pelo Parlamento.

6 Nesta e nas demais citações em língua estrangeira a tradução é livre.

7 Os partidos *Whig* e *Tory* derivam de posições assumidas nas crises que colocaram em lados opostos Parlamento e Coroa desde o século XVII. Tradicionalmente, os *whigs* se identificavam com a defesa da contenção do poder real e a autoridade e independência do Parlamento em relação à monarquia. Eram mais simpáticos à tolerância religiosa e, no século XIX, os mais alinhados com as burguesias industriais e urbanas. *Tories*, por sua vez, enfatizavam a potestade do Trono e da Igreja Anglicana, embora reconhecessem que se deveria respeitar os termos estabelecidos com a Revolução Gloriosa. Em 1835, reestruturaram-se para formar o Partido Conservador.

8 No final do reinado de Elizabeth, em 1601, as mais de duas dúzias de estatutos promulgados ao longo da Dinastia Tudor referentes à pobreza e à vadiagem foram compilados em um código coeso, conhecido como Lei dos Pobres (*Poor Law*). A legislação empoderou as paróquias para arrecadar impostos locais e eleger autoridades com a finalidade de prestar assistência aos pobres que demandassem socorro público, pelos quais os poderes paroquianos eram responsáveis. Para uma introdução às origens dessa instituição, ver Slack (1990).

9 Sobre a interpretação equivocada dos reformadores sobre o pauperismo, ver Boyer (1990). A respeito das continuidades e rupturas no exercício da Lei dos Pobres após a reforma, conferir, além deste último, Englander (2013) e Green (2010).

10
Fraser (1984, p.89-94) chega a considerar a nova Lei dos Pobres o embrião do Serviço Nacional de Saúde.

11 Expressão pela qual eram conhecidos os relatórios de inquérito apresentados ao Parlamento, uma vez que recebiam encadernação azul.

12 A respeito das diferentes formações e ocupações que compunham o campo da medicina e como se deu a regulamentação para definir os contornos de cada profissão da saúde no século XIX, ver Kanikadan e Marques (2013).

13 O desejo de manter os proprietários diretamente envolvidos na gestão do pauperismo transparece na justificativa dos comissários para optar pela manutenção de impostos locais, rejeitando a proposta de um imposto nacional para a Lei dos Pobres (Great Britain, 1834, p.99-100). Sobre as formas pelas quais o ato de emenda de 1834 elevou o controle dos proprietários sobre a Lei dos Pobres, ver Boyer (1990, p.200-201).

14 Para balanços sobre a realidade de trabalho infantil em perspectiva ampla durante a Revolução Industrial na Inglaterra, ver Kirby (2003) e Humphries (2010). Sobre a condição de saúde das crianças envolvidas com o trabalho na indústria, ver Kirby (2013).

15 Sobre a repercussão das ideias de Malthus na comunidade médica, ver Hamlim e Gallagher-Kamper (2000).

16 O parágrafo, publicado pelo economista ricardiano escocês Ramsey McCulloch no periódico liberal *The Edinburgh Review*, foi incluído por Kay em seu relato de 1832.

17 Casa onde vive o trabalhador rural.
